# Shared Investment Projects and Forecasting Errors: Setting Framework Conditions for Coordination and Sequencing Data Quality Activities

**DOI:** 10.1371/journal.pone.0121362

**Published:** 2015-03-24

**Authors:** Stephan Leitner, Alexander Brauneis, Alexandra Rausch

**Affiliations:** 1 Faculty of Management and Economics, Institute of Business Management, Alpen-Adria Universität, Klagenfurt, Austria; 2 Faculty of Management and Economics, Institute of Finance, Alpen-Adria Universität, Klagenfurt, Austria; Politehnica University of Bucharest, ROMANIA

## Abstract

In this paper, we investigate the impact of inaccurate forecasting on the coordination of distributed investment decisions. In particular, by setting up a computational multi-agent model of a stylized firm, we investigate the case of investment opportunities that are mutually carried out by organizational departments. The forecasts of concern pertain to the initial amount of money necessary to launch and operate an investment opportunity, to the expected intertemporal distribution of cash flows, and the departments’ efficiency in operating the investment opportunity at hand. We propose a budget allocation mechanism for coordinating such distributed decisions The paper provides guidance on how to set framework conditions, in terms of the number of investment opportunities considered in one round of funding and the number of departments operating one investment opportunity, so that the coordination mechanism is highly robust to forecasting errors. Furthermore, we show that—in some setups—a certain extent of misforecasting is desirable from the firm’s point of view as it supports the achievement of the corporate objective of value maximization. We then address the question of how to improve forecasting quality in the best possible way, and provide policy advice on how to sequence activities for improving forecasting quality so that the robustness of the coordination mechanism to errors increases in the best possible way. At the same time, we show that wrong decisions regarding the sequencing can lead to a decrease in robustness. Finally, we conduct a comprehensive sensitivity analysis and prove that—in particular for relatively good forecasters—most of our results are robust to changes in setting the parameters of our multi-agent simulation model.

## Introduction

Capital budgeting and, in particular, ensuring the efficiency of investment decisions are among the most important tasks of corporate financial management [1–3]. In corporate practice, it can be observed that organizations with decentralized and distributed decision making authority are very common—in particular with respect to investment decisions. This is due, amongst others, to constantly changing markets, products, or technologies [4], and, in many cases, better informed decentralized units, as compared to the central unit, with respect to these rapidly changing conditions (e.g., [5]). Thus, organizational decentral units can usually evaluate investment opportunities on a more sound knowledge basis. In this context investment decisions are rather complex because these investment decisions often need to be coordinated across multiple departments. Moreover, projects may require to be operated jointly by two or more departments and the managers’ interests are often divergent across these departments. We particularly focus on such scenarios, where investment opportunities are operated jointly by at least two departments. We will, henceforth, refer to such projects as joint or shared investment projects. Furthermore, since firms’ budgets are usually limited, multiple investment opportunities may compete for the same (limited) amount of financial resources with decision externalities as a result [6, 7]. The key issue of coordinating such distributed decisions is to allocate scarce financial resources so that the firm’s profit maximizes. Value creating firms do so not for their own but for their shareholders’ sake—for the purpose of long-term value creation [8]. In order to cope with the complex task of coordinating distributed investment decisions and prevent managers from failing to maximize total profits [6, 7, 9, 10], well developed ‘decision making rules and procedures’ have turned out to be essential. When the department managers are endowed with investment decision making authority [9], decision making rules and procedures help to hold up the efficiency of investment decisions (in terms of value maximization), and to align the department managers’ decisions with a firm’s overall objective of value maximization [7, 10–12]. Such rules and procedures are typically embodied in coordination mechanisms. Given that coordination mechanisms are appropriately set, there should be no loss in efficiency from the firm’s point of view, but distributed investment decisions should be coordinated in the most efficient way.

Coordination mechanisms to ensure efficient distributed investment decisions often utilize so-called ‘hurdle rates’. Hurdle rates are usually referred to as the required rates of return that an investment opportunity has to earn [3]. Since distributed decision making also brings along an increased relevance of performance assessment within the organizational units [9], hurdle rates can help by specifying the capital charge for opportunity costs of capital, which departments get charged. The cost of capital is then subtracted from operating income in order to compute the departments’ performance measures, namely residual income (RI) [2, 7]. The idea of hurdle rates appears to be related to the concept of transfer prices in the sense that the hurdle rate can be regarded as the particular price that a department gets charged for the invested amount of money. The standard textbook solution recommends equating the hurdle rate with the firm’s costs of capital [3, 8]. For unlimited financial resources (or at least for scenarios where the required money to operate investment opportunities is covered by the available resources), this appears to be a plausible approach [6, 13]. However, this does not provide a solution for the case of distributed decisions in combination with the operationalization of multiple competing shared investment opportunities against the background of limited financial resources.

Baldenius, Dutta, and Reichelstein [7] take account of this and introduce a concept of a hurdle rate based coordination mechanism. Baldenius, Dutta, and Reichelstein’s mechanism considers investment opportunities, which are mutually exclusive due to scarce financial resources. The hurdle rate based mechanism is a capital budgeting mechanism for coordinating distributed investment decisions according to which a cost charge, which departments get charged in every subsequent time period in the case that they decide to operate an investment opportunity, is primarily based on divisional reports about the projects’ characteristics. These cost charges comprise depreciation and capital charges (see [7, 14]), whereby the capital charges are based on hurdle rates, which are competitively (and similarly to a second price auction, see [15]) computed on the basis of all divisional reports. This mechanism is derived from an agency model and is shown to be strongly incentive compatible as long as assumptions regarding the involved individuals and the information accessible to the individuals are met. Agency models like this one usually build on a couple of core assumptions such as the existence of fully rational, perfectly homogenous, and non interacting agents. Axtell [16] and Guerrero and Axtell [17] refer to these core assumptions as the ‘neoclassical sweetspot’. In most cases, these assumptions are made in order to assure analytical tractability [18, 19]. Hendry [20] reasons another feature, which is usually incorporated into agency models, i.e., the agents’ full competence in carrying out tasks. However, given this large set of assumptions, it is reasonable to suggest that the agency models’ outcomes are sensitive to changes in their basic frameworks.

Leitner and Behrens [10, 21] abstain from the abovementioned assumptions incorporated in the model from which the hurdle rate based coordination mechanism is derived and go beyond the ‘neoclassical sweetspot’. Hence, they assume away full rationality, perfect homogeneity (regarding departments as well as regarding investment opportunities), and the department managers’ perfect competence in forecasting. By doing so, they investigate the robustness and applicability of such a mechanism to real world situations beyond the strict neoclassical assumptions. Notwithstanding, Leitner and Behrens [10, 21] hold up the assumption that projects are carried out by exactly one organizational unit while simply assuming away spillover effects. We follow this line of research and adopt the mechanism proposed by Baldenius, Dutta, and Reichelstein [7] and Leitner and Behrens [10, 21] to situations in which decisions regarding shared investment opportunities, i.e., they are carried out by ≥ 2 departments, are to be coordinated so that the firm reaches the efficient frontier. This extension is highly relevant, as there is empirical evidence that cooperation among departments is steadily increasing (cf. [22]), which inevitably brings along the need to jointly operate projects. To the best of our knowledge, there is no such mechanism existent in the literature, which fulfills strong incentive compatibility. Please note that Baldenius, Dutta, and Reichelstein [7] provide a coordination mechanism for (i) exclusively carried out and competing investment projects. Leitner and Behrens [10, 23] transfer this mechanism into a multi-agent model but hold up the assumption that one project is carried out by exactly one department. Moreover, Baldenius, Dutta, and Reichelstein [7] propose a mechanism for (ii) jointly carried out investment projects, which does not consider competing projects, but only focuses on the allocation of resources given that one common asset is acquired. Thus, there is no coordination mechanism, which focuses on the coordination of multiple competing and mutually carried out investment projects. The work of Baldenius, Dutta, and Reichelstein [7] and Leitner and Behrens [10, 21], as well as the way their work relates to our model, will be discussed in more detail in Sect. ‘Rendering agency models into computational multi-agent models’. We, then, on the basis of the transferred mechanism, build a simulation model and relax crucial assumptions, which are typically embodied in agency models (e.g., assumptions regarding information sharing, perfect foresight). By doing so, we follow the so-called ‘agentization’-approach [23]. As suggested by Leitner and Behrens [10, 21], we particularly take into account limited rationality, heterogeneity with respect to investment opportunities and departments, and a certain level of incompetence in forecasting measures associated with investment opportunities. Relaxing the crucial assumption of perfect information allows for investigating the efficiency of our proposed mechanism to errors in forecasting measures associated with investment opportunities. As a consequence, in this paper we particularly focus on two research questions: First, we are particularly interested in the framework conditions, that make our proposed mechanism highly robust to such errors, given that all measures associated with investment projects are forecasted with error. By doing so, we contribute to the existing literature on the coordination of distributed investment decisions, since we relax intra-organisational boundaries and allow collaboration among departments. By comparison to the case of efficiently made investment decisions (in terms of value maximization), we narrowly characterize efficiency losses. Second, we are interested in the sequencing of data quality activities, i.e., in the order in which organizations should learn to forecast measures associated with investment projects, whereby learning to forecast is associated with costs (e.g., for training). In other words, we aim at providing guidance on where to invest resources in order to increase forecasting accuracy, so that investment for increasing forecasting quality are highly efficient, i.e., their outcome has the best possible impact on the efficiency of budget allocation and value creation in terms of shareholder value (SHV). This also is a particular novel aspect in this line of research, as neither [10] nor [21] focus on simultaneously made errors and on sequencing investments in order to increase forecasting quality, but only report the effect of such errors in isolation. Furthermore, we conduct a comprehensive sensitivity analysis, which allows for insights into the dynamics of our mechanism to coordinate autonomous and decentralized decisions regarding jointly carried out investment projects.

Since hurdle rate based as well as transfer price based coordination mechanisms seem to be frequently utilized in corporate practice [7], the investigation of the robustness of such mechanisms appears highly relevant, not least from a practitioner’s point of view. In addition, our work complements previous research on hurdle rates in the context of corporate investment decisions, including Dutta and Reichelstein [24], Baldenius, Dutta, and Reichelstein [7], and Baldenius [2], as well as work on the robustness of such coordination mechanisms to situations that are closer to real world situations [10, 21]. We investigate scenarios where a coordinating unit (e.g., a firm’s central office) announces project specific costs of capital in response to department managers’ reports regarding investment opportunities. Our mechanism is a direct mechanism because the managers report private information (i.e., estimations) about the required amount of money necessary to launch the project, the intertemporal distribution of cash flows, and the departments’ efficiency in operating the investment opportunity directly to the coordinating unit. This brings along the advantage that department managers have to evaluate a manageable number of investment opportunities [6]. Departments have decision making authority, but no financial autonomy. Consequently, they must rely on funding from the coordinating unit. However, due to scarce financial resources, only a subset of the proposed investment opportunities can ultimately be funded. The departments autonomously decide whether investment opportunities are put into practice. This decision is aligned with the organizational objective employing a capital charge rate: The coordinating unit computes a specific hurdle rate for each investment opportunity, whereby the hurdle rate serves as the basis for the cost charges imposed on the departments in subsequent periods. Utilizing the relative benefit depreciation rule [14], we model the departments’ performance measure so that—in the case of putting it into practice—the projects’ net present values (NPV) are reflected in the departments’ performance measure in a time-independent manner.

In order to learn about the robustness of the proposed mechanism to coordinate distributed decisions regarding the operationalization of joint investment opportunities, we employ a multi-agent simulation approach. Doing so allows for investigating how micro-level actions, i.e., the actions taken by department managers, affect the macro-level outcome, i.e., the extent to which the firm reaches the efficient frontier in terms of SHV maximization [25]. Employing a multi-agent simulation approach has several advantages over empirical and formal research methods. Investigating the research question with experimental methods would be particularly difficult because—in an experiment—it cannot be assured that the variables under research are free from the influence of other factors such as cognitive biases [26, 27]. Employing a multi-agent simulation, conversely, allows for disentangling such effects [28–31]. Formal modeling approaches might also find their boundaries due to the complexity of the research questions contained in our agenda. It is well known that repeated simple patterns can lead to complex situations, which cannot easily be traced if formal methods are employed [29, 32]. When decisions regarding the operations of shared investment projects are to be coordinated, when the involved managers’ decisions are potentially divergent, and when the investment opportunities are competing for scarce financial resources, it is very likely that a level of complexity is reached, which does not allow for tracing by formal methods. Multi-agent simulations, however, appear to be a powerful method to overcome the weaknesses outlined above and to tackle the complexity of the questions contained in the research agenda [33].

The remainder of the paper is structured as follows: in Sect. ‘Rendering agency models into computational multi-agent models’ we elaborate on the process of transferring the basic idea of an agency model into a multi-agent model, introduce necessary adaptions of the mechanism proposed by [7] (i.e., in particular the allocation mechanism for the money necessary to launch an investment opportunity), formally describe the simulation model, and introduce the measures utilized in our data analysis. Sect. ‘Simulation results’ introduces the results of our simulation experiments. In order to illustrate the functioning of the mechanism, we start by introducing an example with perfect foresight. We, then, add uncertainty (i.e., all measures associated with investment opportunities are erroneous), and we show that losses can be minimized (and, thus, the efficiency of the coordination mechanism can be increased) if only a few projects are considered in one round of budget allocation. We also define certain setups, in which a certain extent of uncertainty even leads to an increase in SHV, as compared to scenarios with full certainty regarding the measures associated with investment projects. In a next step, we again add certainty regarding particular measures associated with investment projects, i.e., we model that the departments learn to perfectly forecast. We show that the number of departments operating one joint project is a crucial policy parameter for controlling the loss measures. However, for organizations, which are unable to alter the extent of cross-departmental cooperation in the short run, we provide guidance on how to sequence data quality activities (i.e., which measure to learn to perfectly forecast with a higher priority, so that the efficiency of the mechanism—and, as a consequence, SHV—increases in the best possible way). We complement our data analysis with an extensive analysis of the sensitivity to important model parameters. In Sect. ‘Summary and policy reflections’ we provide a summary and a discussion of our results as well as policy reflections and an outlook on potential avenues for future research.

## Rendering agency models into computational multi-agent models

**Fig 1:**
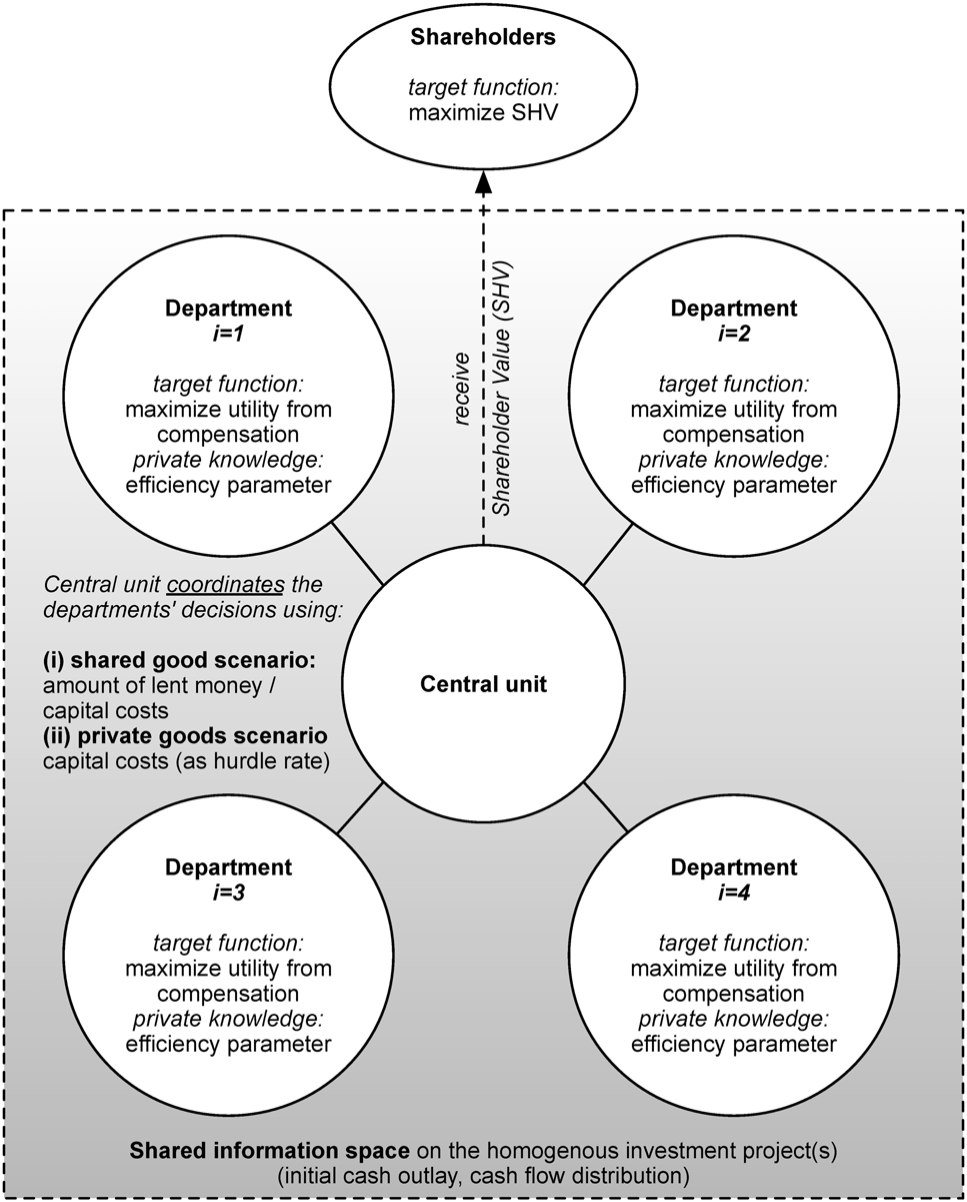
Schematic representation of the basic model proposed by Baldenius, Dutta, and Reichelstein [7] (for four departments).

In their seminal paper, Baldenius, Dutta, and Reichelstein [7] propose an agency model from which a coordination mechanism for distributed investment decisions is derived. On the basis of the model proposed in [7], we build a multi-agent computational model. Our approach is to render neoclassical models into computational multi-agent models. This allows for investigating how modifying (sometimes rather restrictive) assumptions incorporated in agency models affects the models’ outcomes, i.e., in our case the efficiency of the proposed coordination mechanism (see also the work done by [10, 11, 17, 21]). Moreover, we focus on the first-best solution proposed in Baldenius, Dutta, and Reichelstein [7]. The second-best solution (i.e., considering untruthful reporting from the departmental side) should be considered in future model variants. Agency models and multi-agent models are two very distinct types of models. Agency models are concerned with problems between two parties to a contract, with different objectives, hidden information, and/or imperfect observation of the agents’ actions on the part of the principal (see [33–35]). Multi-agent models, on the contrary, aim at analyzing the impact of agents’ actions on the micro-level (i.e., the departmental level) and on the outcome on the macro-level (i.e., the corporate level, e.g., in terms of efficient project selection and implementation) (see [25, 36]). The model presented here is very close to an agent-based model. According to Epstein [37], agent-based models are characterized by (i) heterogeneous agents, (ii) autonomously acting agents, (iii) an explicit space over which agents interact, (iv) local interactions, and (v) bounded rational agents. As will be outlined in Sect. ‘Computational representation of the multi-agent model’, the multi-agent model presented in this paper fulfills the majority of the characteristics elaborated in [37]. In our model variant, agents are bounded rational, autonomously acting, and heterogeneous. We have designed the interaction in a way that makes departments locally (and *indirectly*) interacting over the coordination mechanism and over an explicit interaction space. As we have decided to incorporate (only) *indirect interaction* between departments, we will henceforth refer to our model variant as a computational representation of a multi-agent model. Moreover, agent-based and multi-agent models usually investigate the adaption of a systems or individuals over time [32, 38, 39]. Typically, agent-based models follow specific design patterns. Prominent modeling approaches for agent-based models are (i) NK-fitness landscapes (cf. [40]), which are frequently utilized for the modeling of adaptive search and optimization processes in modular systems, (ii) genetic algorithms (cf. [41]), which are mainly used to model learning of a population of heterogeneous agents, and (iii) cellular automata (cf. [42]), which are mainly concerned with spatially related agents and diffusion processes. Our simulation approach does not focus on adaption, learning, or diffusion processes, but investigates the efficiency of the proposed mechanism in a setup where information involves a certain extent of uncertainty. We do not model agents to make decisions over time (as usually considered by the dynamic approaches (i) to (iii)), but investigate the efficiency of a decision in a static environment. Consequently, according to Davis, Eisenhardt, and Bingham [32] and Deckert and Klein [38], our approach can be regarded as a static stochastic modeling approach. In this section, we give an overview of the agency model introduced in [7] which we, henceforth, refer to as the ‘basic model’ (see Fig. 1). Moreover, we elaborate on the main assumptions contained in the basic model and discuss which of these assumptions are relaxed in order to build our variant of a multi-agent model.

The basic model distinguishes between two scenarios, namely the *(i) shared good* and the *(ii) private good scenario*. For both scenarios, the organizations under investigation are comprised of a coordinating unit (i.e., the CEO or principal, respectively) and a number of departments (i.e., the agents in terms of an agency model). Departments have decision making authority with respect to whether to operate a project, but no financial autonomy. Hence, they cannot acquire financial resources from outside the firm but must rely on funding from the coordinating unit. The departments and the CEO are assumed to have different time preferences with the result that there are different planning horizons. All departments are assumed to be rational and fully competent to forecast all measures associated with investment projects without errors. Since the CEO faces scarce financial resources, only one investment project can be funded at a time. In order to derive maximum utility, departments aim at maximizing their variable compensation, which is a function of residual income, *f*(*RI*). As the main shareholders’ claim is wealth maximization, the principal aims at maximizing SHV. SHV is defined by the operated project’s NPV minus the agents’ variable compensation, i.e., *NPV* − *f*(*RI*). Regarding the availability of information, the basic model assumes all parties (i.e., the CEO as well as all departments) to have a shared information space, which contains measures associated with investment projects (i.e., initial cash outlay, inter temporal distribution of cash flows). Moreover, each department is characterized by an efficiency in operating investment projects. The knowledge about the efficiency is assumed to be the departments’ private knowledge.The following paragraphs outline the basic ideas behind the mechanisms proposed by Baldenius, Dutta, and Reichelstein [7] in a nutshell. The *(i) shared good scenario* captures situations in which *one* common asset is acquired to which all departments have access. For such situations, the so-called Pay-the-Minimum-Necessary (PMN) mechanism is derived from the basic model. The PMN mechanism aims at efficiently allocating the money necessary to launch the joint project and the associated capital costs resulting from the borrowed money in order to allow for efficient project selection (or participation) decisions. In the *(ii) private good scenario*, the basic model assumes each department to have an investment opportunity and the investment alternatives not to differ in their associated measures (namely the initial cash outlay and the intertemporal distribution of cash flows). As, at most, the operationalization of one investment opportunity can be funded, all departments compete for the scarce financial resources. For this setup, the competitive hurdle rate (CHR) mechanism is derived from the basic model (see Fig. 1). The CHR mechanism provides departments with incentives so that they align their decisions to the firm’s overall objective of SHV maximization. In a nutshell, coordination on the basis of the CHR mechanism is driven by the interest rates, which departments get charged if they decide to borrow money from the coordinating unit in order to operationalize an investment opportunity.

While the (i) *shared good scenario* does not include coordination of multiple competing projects but only focuses on the efficient budget allocation, the (ii) *private good scenario* focuses on the efficient coordination of decisions regarding the operationalization of investment opportunities where each opportunity is proposed by exactly *one* department. In their paper, Leitner and Behrens [10] provide a multi-agent model of the (ii) private good scenario. Please note that a version of the model utilized in [10] is also utilized in [21]. In both cases ([10] and [21]) Leitner and Behrens deploy the idea of a hurdle rate inspired coordination mechanism to situations, in which the involved individuals make forecasting errors. In contrast, in [23], Leitner and Behrens use their model variant in a setup in which the involved individuals are fully competent to forecast without any error (while all other modifications incorporated in [23] (as compared to Baldenius, Dutta, and Reichelstein [7]) are in line with [10] (cf. also Table 1)). In [23], Leitner and Behrens extensively elaborate on the dynamics of the hurdle rate inspired coordination mechanism, while in [10] and [21], they focus on the impact of errors on the robustness of the proposed mechanism. In particular, they transfer the idea behind the CHR mechanism into a simulation model and investigate the impact of forecasting errors on the mechanism’s efficiency for the case of heterogeneous investment opportunities. In this paper, we investigate a setting in which a number of joint investment opportunities (i.e., investment opportunities, which are submitted as a proposal to the coordinating unit by ≥ 2 departments) compete for scarce financial resources. As the (ii) *private good scenario* explicitly includes coordination, we follow the approach of [10] and utilize this scenario as the basis for our model variant. Our aims are twofold. First, we are interested in adapting the private good scenario to cases where departments apply for funding for investment opportunities, which are carried out mutually. This means that we extend the model proposed by [10] in two ways: We allow for interdependencies among departments, and extend the CHR inspired mechanism proposed in [10] by an allocation rule for the cash necessary to launch the investment project. We further test the impact of these two adaptions on our coordination mechanism’s efficiency. Second, we aim at relaxing some of the assumptions incorporated in the basic model (see Fig. 1), and we aim at further developing the model variant proposed in [10]. Table 1 provides an overview of the main assumptions incorporated in the basic model, the model utilized by Leitner and Behrens [10], and our model variant.

**Table 1:** Relevant assumptions within the basic model [7], the model variant of Leitner and Behrens [10], and our model variant.

No.	Basic model	Model variant of [10]	Our model variant
**Principal (coordinating unit) …**			
1	Faces scarce financial resources	Carried over	Carried over
2	Aims at maximizing shareholder value	Carried over	Carried over
**Agents (departments) …**			
3	Are heterogeneous in their efficiencies of operating projects	Carried over	Carried over
4	Have time preferences, which potentially differ from the firm’s time-horizon	Carried over	Carried over
5	Are rational in decision making	Carried over	Carried over
6	Are competent to forecast measures associated with investment projects free of errors	Forecasting errors are added	We add forecasting errors
7	Have decision making authority regarding whether or not to operate a project	Carried over	Carried over; in contrast to [7] and [10], investment opportunities are only put into action if and only if all departments involved in one investment opportunity decide to operate the project
8	Do not have financial autonomy	Carried over	Carried over
**Investment alternatives …**			
9	Are proposed by exactly *one* department	Carried over	Are proposed by *z* ≥ 2 departments
10	Are homogeneous (with respect to initial cash outlay, return on investment, and intertemporal cash flow distribution)	Are heterogeneous (with respect to all measures associated with investment projects)	Are heterogeneous (with respect to all measures associated with investment projects)
**Information spaces:**			
11	One shared information space, which contains all measures associated with investment projects	Project-specific knowledge is available only for the corresponding departments, i.e., information spaces are disjointed	Disjointed information spaces: (i) for each project *j* a information space is shared by *z* departments, (ii) one information space for the coordinating unit
12	Private knowledge of the departmental efficiency parameter	Carried over	Carried over

**Fig 2:**
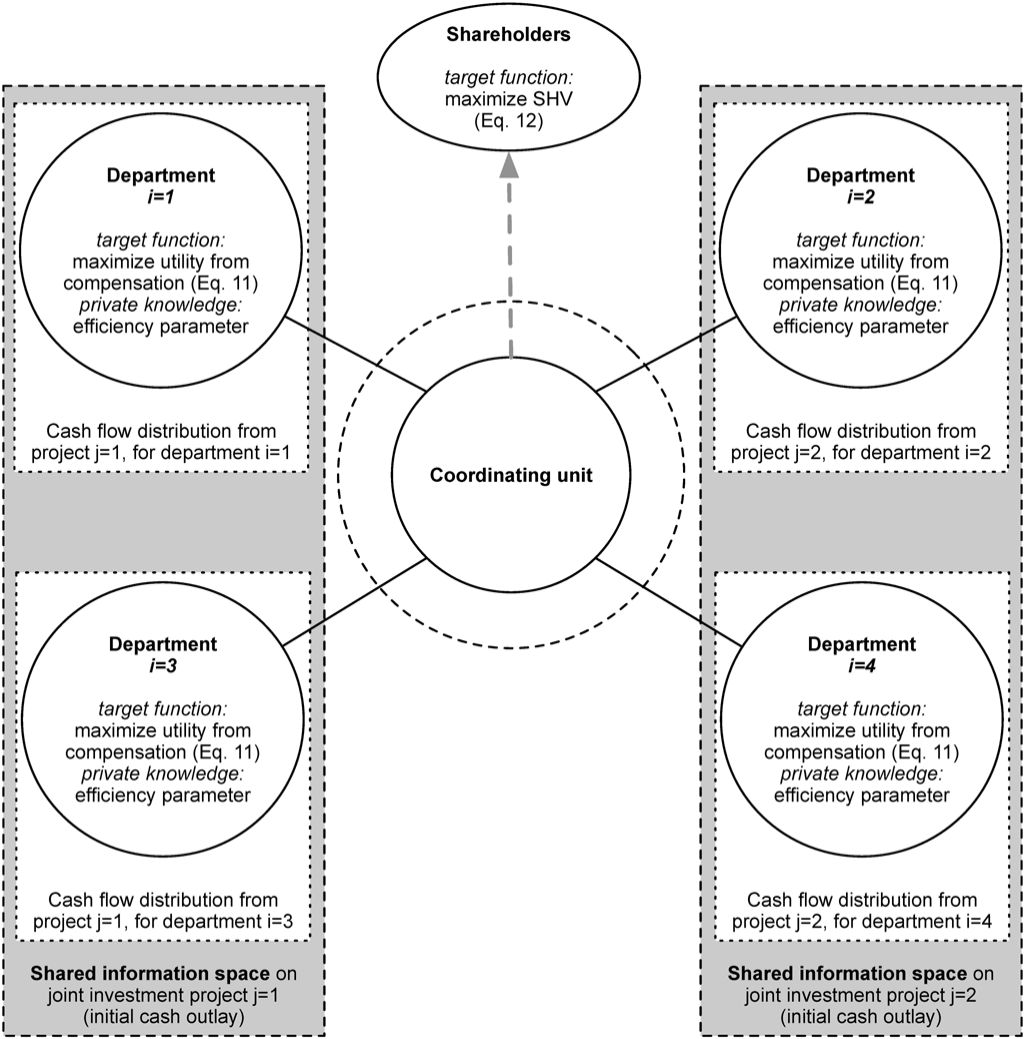
Structure of our model variant (for four departments and two joint projects).

The structure of our model variant is exemplarily depicted in Fig. 2 (for four departments and two joint investment opportunities, each proposed by two departments). Regarding the CEO, we carry over all assumptions from [7], but we limit the departments’ foresight in forecasting the measures associated with investment opportunities as suggested by [10]. Thus, in addition to the heterogeneity of the departments with respect to their efficiency in operating investment projects, we model heterogeneity also with respect to the departments’ accuracy in forecasting (see Table 1, assumption 6). By contrast, we relax the homogeneity assumption with respect to the characteristics of investment opportunities. Thus, contrary to [7] but in line with [10], we assume the investment opportunities, which are submitted to the coordinating unit to be heterogeneous with respect to their initial cash outlay and the intertemporal distribution of cash flows (see Table 1, assumption 10). Contrary to [7] and [10], we model investment opportunities to be proposed to the coordinating unit by ≥ 2 departments. (see Table 1, assumption 9). Each department taking part in the operationalization of an investment opportunity generates cash flows from this very project. For all investment opportunities, we make the associated information regarding the intertemporal distribution of cash flows and the departments’ efficiency parameters for operating investment opportunities the involved departments’ private information, thereby creating disjointed information spaces (see Table 1, assumption 11). As a consequence of modifying assumption 11, we differ from both [7] and [10] in the modeled information spaces. While [7] assume one information space which is shared by all departments and the coordinating unit, [10] model disjointed information spaces for all departments as well as the coordinating unit. As knowledge on measures associated with investment opportunities, in most cases, is gained through forecasting and the departments are usually better informed about their projects than the coordinating unit or the other departments, this is a feasible assumption. All departments involved in one project proposal share one information space, which contains the project’s initial cash outlay. In Fig. 2, the information spaces regarding the joint projects are indicated by dashed boxes. Please note that, with respect to the model, we do not only differ from Leitner and Behrens [10] in modifying assumptions 9 and 11 (cf. Table 1), but also in the coordination mechanism itself. In order to deploy the mechanism to situations, in which investment opportunities are proposed jointly by departments, it needs to be extended by an allocation rule for the initial amount of money necessary to launch the investment project (for the allocation rule cf. Equation 7, for the consequences of this adaption cf. Equations 8 to 10). Moreover, as will be outlined in Sect. ‘Simulation Setup’, we also differ in some basic model features (i.e., amongst others we differ in the stochastic processes for generating the intertemporal distribution of cash flows and the approach for generating the amount of money necessary to launch projects).

**Fig 3:**
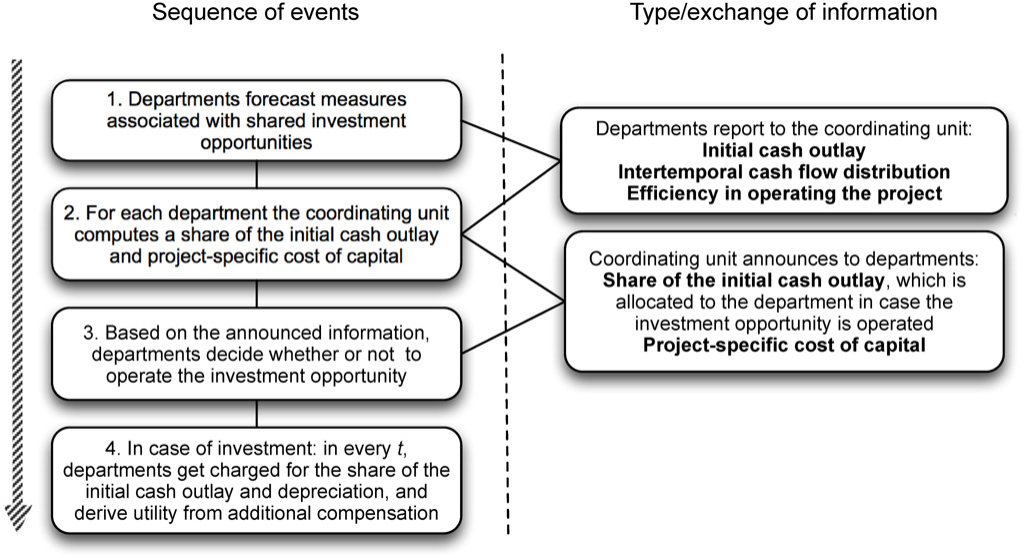
Sequence of events.

We exclude the process of forming cooperations for proposing joint projects from this investigation, but focus on the efficiency of our hurdle rate inspired mechanism. The coordination mechanism for jointly proposed projects is implemented as follows (see also Fig. 3): All departments forecast the measures associated with investment projects, i.e., the initial cash outlay (for the entire project), the distribution of cash flows, and their efficiency in operating the joint project. This information is forwarded to the coordinating unit, which (i) computes project specific costs of capital, and (ii) decides how the initial cash outlay is allocated across the involved departments. Recall that we emanate from the assumption of scarce financial resources, which is why the coordinating unit fixes the capital charge rates and the fractions of initial cash outlay so that only the department which proposes the highest NPV project is provided with incentives to operate the project. Then, information on the capital costs in terms of an interest rate and the assigned fraction of initial cash outlay is returned to the departments, before the final investment decisions are made. Considering future utility, departments decide on the basis of this information whether or not to operate the investment opportunity. If proposed projects are put into action, the involved departments get charged for the borrowed money throughout the investment’s entire lifetime (i.e., from their cash flows they have to pay depreciation and capital charges to the coordinating unit), and in every time period they derive utility from the residual income of the operated investment opportunity.

### Computational representation of the multi-agent model

We model firms that consist of *m* (*i* = 1, …, *m*) departments and one coordinating unit. Regarding investment decisions, we endow departments with decision making authority with respect to whether or not to operate investment opportunities. However, departments do not have financial autonomy, and, consequentially, departments cannot acquire financial resources from outside the organization. Whenever they want to operate an investment opportunity they have to apply for funding from the coordinating unit.

#### A feasible investment strategy

At time period *t* = 0, departments are in charge of proposing shared investment projects, *n* (*j* = 1, …, *n*), to be realized in the following time periods, *t* (*t* = 1, …, *T*), where each project is carried out by *z* departments and each department can only participate in one joint investment project. Thus, *z* = *m*/*n* ≥ 2. The function *f*(*i*, *j*) ∈ {0, 1} expresses whether department *i* is involved in project *j* (i.e., *f*(*i*, *j*) = 1) or not (i.e., *f*(*i*, *j*) = 0). In turn, for the application for funding, the coordinating unit announces a capital charge rate that the departments get charged for the initially borrowed money, if they decide to operate their proposed project. This autonomous decision is denoted by the binary variable *I*_*ij*_ ∈ {0, 1} that indicates if department *i* decides to operate project *j* (*I*_*ij*_ = 1) or not (*I*_*ij*_ = 0). The coordinating unit faces the problem of limited financial resources, so that, at most, one investment opportunity at a time can be funded. Thus, a feasible investment strategy fulfills

$$\begin{array}{c} \frac{\sum_{i=1}^{m}\sum_{j=1}^{n}I_{ij}}{z}=1, \end{array}$$ (1)

subject to $\forall j:\sum_{i=1}^{m}I_{ij}\in \{0,z\}$ and $\forall i:\sum_{j=1}^{n}f\left(i,j\right)=1$. This assumption implies that a project is carried out by exactly *z* departments. Thus, if project *j* gets realized, *I*_*ij*_ = 1∀*i*:*f*(*i*, *j*) = 1. An efficient allocation of the firm’s limited financial resources induces SHV maximization, which comprises the firm’s main objective (see also [8, 43]). Thus, an efficient allocation mechanism needs to provide departments with incentives to put the investment opportunity that yields the maximum increase in SHV into action.

#### Measures associated with investment projects

At the beginning of the allocation procedure, at *t* = 0, we normalize all status quo cash flows to 0. Let each investment opportunity, *j*, be characterized by an initial cash outlay, *κ*_*j*_, necessary to launch the project. For each department *i* involved in project *j* there is an intertemporal distribution of cash flows denoted by the *T*-dimensional row vector **x**_*ij*_ = [*x*_*ij*1_ *x*_*ij*2_ … *x*_*ijT*_]. For each department, **x**_*ij*_ is private information (see the dotted lines in Fig. 2). Finally, each department is characterized by the parameter *ρ*_*ij*_ that captures department *i*’s efficiency in operating the jointly proposed project *j*. The parameter *ρ*_*ij*_ scales the generated cash flows. Thus, department *i*’s operative cash flow generated from project *j* in time period *t* results in *x*_*ijt*_ ⋅ *ρ*_*ij*_.

As all measures related to investment projects are reported to the coordinating unit, the coordinating unit’s information space (see the dashed circle in Fig. 2) is filled. The coordinating unit, in turn, computes and announces capital charge rates (see Sect. ‘Computation of the project specific costs of capital’) and a key for allocating the initial cash outlay across departments (see Sect. ‘Allocation of the initial cash outlay’).

#### Computation of the project specific costs of capital

In order to compute the capital costs for each project and each department, the coordinating unit starts with calculating the project’s PV. We denote the discount factors necessary to discount future cash flows by the column vector **r**(*r*) = [(1+*r*)^−1^ … (1+*r*)^−*T*^]^′^, where *r* stands for the costs of capital in terms of an interest rate and the prime indicates transpose. The PV of department *i*’s cash flows generated from project *j* results in

$$\begin{array}{c} PV_{ij}\left(x_{ij},\rho_{ij},r_{}\right)≔x_{ij}\circ r\;\left(r_{}\right)\cdot \rho_{ij}\;. \end{array}$$ (2)

Consequentially, project *j*’s NPV results in

$$\begin{array}{c} \Lambda_{j}\left(r,p_{j}\right)≔\sum_{\forall i:f\left(i,j\right)=1}{PV}_{ij}\left(x_{ij},\rho_{ij},r_{}\right)-\kappa_{j}\;, \end{array}$$ (3)

where **p**_*j*_ is a *z*-dimensional row vector containing the efficiency parameters, *ρ*_*ij*_, of all departments, which are involved in project *j*, i.e., ${\text{p}}_{j}=[\rho_{ij}^{1}\;...\;\rho_{ij}^{z}],\;\forall i:f\left(i,j\right)=1$. Augmenting Equations 2 and 3 by the firm’s costs of capital, *r*_*c*_, yields that the NPVs for projects {1, …, *n*} are given by Λ_1_(*r*_*c*_,**p**_1_), …, Λ_*n*_(*r*_*c*_,**p**_*n*_). Next, out of the NPVs associated with proposals, a reference NPV, i.e., the highest NPV of all projects other than *j*, is computed for each project *j*. We formalize the reference NPV, $\Lambda_{j}^{*}$, as follows

$$\begin{array}{cc} \Lambda_{j}^{✱}=max\{ & \Lambda_{1}\left(r_{c},p_{1}\right),...,\Lambda_{i-1}\left(r_{c},p_{j-1}\right),\\ & \Lambda_{j+1}\left(r_{c},p_{i+1}\right),...,\Lambda_{n}\left(r_{c},p_{n}\right)\}\;. \end{array}$$ (4)

The hurdle rate based coordination mechanism is designed in a similar way to a second-price auction [15] because the project with the highest NPV gets charged on the basis of the project with the second-highest NPV [7]. The highest NPV project is, henceforth, denoted by 𝕛. In order to implement the mechanism in this way, for each department the coordinating unit computes a critical efficiency parameter, $\rho_{ij}^{*}$. The vector containing the critical efficiency parameters of departments involved in project *j* results in

pj✱:=[ρij1✱...ρijz✱]=Λj✱+κjΛjrc,pj+κj︸<1∀j=𝕛>1∀j≠𝕛·pj, (5)

where the fraction $(\Lambda_{j}^{*}+\kappa_{j})/(\Lambda_{j}\left(r_{c},{\text{p}}_{j}\right)+\kappa_{j})$ is < 1 only for the highest NPV project, 𝕛. Thus, the multiplication with this scalar diminishes the efficiency parameters contained in vector ${\text{p}}_{𝕛}^{}$. Then, augmenting Equation 3 by ${\text{p}}_{𝕛}^{*}$, project 𝕛 is as profitable as the second-highest NPV project. To see this, note that the denominator of Equation 5 equals the PV of project *j*, denoted by *PV*_*j*_. Thus, we may rewrite the NPV of project *j* under ${\text{p}}_{j}^{*}$ as $PV_{j}\cdot \frac{\Lambda_{j}^{*}+\kappa_{j}}{PV_{j}}-\kappa_{j}$. This expression reduces to $\Lambda_{j}^{*}$ and, hence, the project’s NPV under the critical efficiency equals the maximum NPV of the remaining projects.

For all projects other than 𝕛, $(\Lambda_{j}^{*}+\kappa_{j})/(\Lambda_{j}\left(r_{c},{\text{p}}_{j}\right)+\kappa_{j})$ is > 1. Thus, the efficiency parameters contained in **p**_*j* ≠ 𝕛_ are increased, so that, in terms of NPV at the critical efficiency, all projects *j* ≠ 𝕛 are as profitable as project 𝕛. Please note, these critical efficiency parameters and the resulting NPVs at this profitability level are *hypothetical values*, which are used in order to compute the hurdle rates $r_{1}^{*},...,r_{j}^{*},...,r_{n}^{*}$. For each project *j*, the hurdle rate, $r_{j}^{*}$, is the internal rate of return at the critical efficiency parameter. Please note that this is the core of our hurdle rate inspired mechanism: The capital charge rate $r_{𝕛}^{*}$ is lower than the project’s internal rate of return only in the case of the winning project, 𝕛. For all other projects *j* ≠ 𝕛, the hurdle rate, $r_{j}^{*}$, is higher than the project’s internal rate of return. Since putting these projects into action would result in a negative NPV (see Equation 3), departments would not opt for these projects. Utilizing the departments’ estimations of the project related measures, the capital charge rates are implicitly defined by solving

$$\begin{array}{c} \Lambda_{j}\left(r_{j}^{✱},p_{j}^{✱}\right)=0\;. \end{array}$$ (6)

#### Allocation of the initial cash outlay

We allocate the initial cash outlay, *κ*_*j*_, which is necessary to operate project *j* relative to the involved departments’ PVs at the critical efficiency parameters. I.e., the share *λ*_*ij*_ of *κ*_*j*_ allocated to department *i* is computed according to

$$\begin{array}{c} \lambda_{ij}=\underset{\text{denominator}\;\text{equals}\;\kappa_{j}}{\underset{︸}{\frac{PV\left(x_{ij},\rho_{ij}^{✱},r_{j}^{✱}\right)}{\sum_{\forall k:f\left(k,j\right)=1}PV\left(x_{kj},\rho_{kj}^{✱},r_{j}^{✱}\right)}}} \end{array}$$ (7)

where the denominator equals project *j*’s initial cash outlay, *κ*_*j*_ (see Equations 3 and 6). This mode of allocating the initial cash outlay among departments yields strong incentive compatibility (see Equation 10). For a definition of strong incentive compatibility see [7], p. 849-851 and p. 862.

#### The departments’ autonomous decision

Next, after computing the hurdle rates, $r_{j}^{*}$, and the capital shares, *λ*_*ij*_, the coordinating unit submits the hurdle rates and the capital shares to the departments. In the next step, departments autonomously decide whether or not to operate their proposed project. Each department’s decision making behavior is described by the following rule

$$\begin{array}{c} I_{ij}=\left\{\begin{array}{cc} 1, & \text{if}\;PV_{ij}\left(x_{ij},\rho_{i},r_{ij}^{✱}\right)-\lambda_{ij}\cdot \kappa_{j}>0\;,\\ 0, & \text{otherwise.} \end{array}\right. \end{array}$$ (8)

according to which departments put only positive NPV projects into action. Thus, operating the investment opportunity is only attractive for departments, which are involved in the highest NPV project 𝕛, i.e., ∀*i*:*f*(*i*, 𝕛) = 1. Whenever departments decide to put their investment opportunities into action, they are charged according to the relative benefit depreciation schedule. We employ the relative benefit depreciation rule in order to overcome differences in the CEOs’ and the departments’ planning horizons (see [14]). Department *i*’s depreciation and capital charge for period *t* results in

$$\begin{array}{c} \zeta_{ijt}=\lambda_{i}\cdot \kappa_{j}\cdot \frac{x_{ijt}}{r\left(r_{i}^{✱}\right)\circ x_{j}}\cdot I_{ij}\;. \end{array}$$ (9)

where **x**_*j*_ denotes the vector of the total cash flows generated by all departments involved in project *j*. In every period *t*, the departments receive a variable compensation as a function of residual income, i.e., *f*(*v*_*ijt*_), where the residual income is comprised of period *t*’s operative cash flow minus the depreciation and capital charge, i.e.,

$$\begin{array}{cc} v_{ijt} & =x_{ijt}\cdot \rho_{ij}-\zeta_{ijt}\\ & =x_{ijt}\cdot \rho_{ij}-\underset{\text{capital}\;\text{charge}\;\zeta_{ijt}\;\text{(Eq.}\;\text{9)}}{\underset{︸}{\underset{\text{share}\;\lambda_{ij}\;(\text{Eq}.\;7)}{\underset{︸}{\frac{PV\left(x_{ij},\rho_{ij}^{✱},r_{j}^{✱}\right)}{\kappa_{j}}}}\cdot \kappa_{j}\cdot \frac{x_{ijt}}{r\left(r_{i}^{✱}\right)\circ x_{i}}}}\;. \end{array}$$ (10)

This performance measure reduces to $v_{ijt}\;=\;x_{ijt}\cdot \rho_{ij}\;-\;x_{ijt}\cdot \rho_{ij}^{*}\;=\;x_{ijt}\left(\rho_{ij}-\rho_{ij}^{*}\right)$, and, thus, fulfills strong incentive compatibility (according to [7]). We denote the sequence of future compensation components by the *T*-dimensional row-vector **v**_*ij*_ = [*f*(*v*_*ij*1_) … *f*(*v*_*ijT*_)]. For simplicity, we assume departments to discount at the firm’s costs of capital. Thus, the departments’ utility function results in

$$\begin{array}{c} U_{ij}\left(r\left(r_{c}\right)\circ v_{ij}\right)\;. \end{array}$$ (11)

Thus, Equation 11 expresses the departments’ aim of maximizing their utility received from the performance measure given by *f*(*v*_*ijt*_). The CEO, however, aims at fulfilling the shareholders’ claim of SHV maximization, which is reflected in the utility function

$$\begin{array}{c} U_{c}\left(\Lambda_{j}\left(r_{c},p_{j}\right)-r\left(r_{c}\right)\circ v_{i}\right)\;. \end{array}$$ (12)

where **v**_*j*_ denotes the vector of the sum of all compensation components assigned to departments operating project *j*.

### Simulation Setup

We model each department *i*’s intertemporal distribution of cash flows **x**_*ij*_ earned from operating project *j* by following a geometric Brownian motion (GBM). This process is also commonly used in modeling financial time series such as stock prices. Please note that we substantially differ from [10] with respect to the stochastic process for generating the intertemporal distribution of cash flows. While [10] describe a stochastic process, which is basically grounded on normally distributed random variables, we rely on the well established GBM process. In particular, we model two correlated cash flow paths (using a bivariate GBM), whereby one corresponds to that actually put into effect and the second is the department’s forecast of cash flows. A process follows a bivariate GBM if it satisfies the stochastic differential equation:

$$\begin{array}{c} dx_{t}^{\left(k\right)}=\mu^{\left(k\right)}x_{t}^{\left(k\right)}dt+\sigma^{\left(k\right)}dB_{t}^{\left(k\right)} \end{array}$$ (13)

where *μ* denotes the drift parameter, *σ* the diffusion rate, *B*_*t*_ a Wiener process and *k* = {*R*, *E*} ‘real’ and estimated values of cash flows. The Wiener processes are correlated such that $\text{E}\left[dB_{t}^{\left(R\right)}dB_{t}^{\left(E\right)}\right]=p_{R,E}dt$ and *p*_*R*, *R*_ = *p*_*E*, *E*_ = 1. We use a zero drift ($\mu_{ij}^{\left(k\right)}=0$) discrete time approximation (using Matlab^©^’s function ‘portsim.m’) of the above process and (arbitrarily) set a constant $\sigma_{ij}^{\left(R\right)}$ for all departments *i* and investment opportunities *j*. Furthermore, *x*_*ijt*_ is normalized to one at *t* = 0. Throughout the whole simulation design, we use the parameter $\sigma_{ij}^{\left(R\right)}=\sigma$ as a general risk parameter causing uncertainty after all.

The diffusion rates of the real and estimated cash flow paths are linked according to $\sigma_{ij}^{\left(E\right)}=\sigma^{\left(E\right)}=\sigma /p_{R,E}$ for all *i* and *j*, with *p*_*R*, *E*_ ∈ (0, 1]. Hence, *p*_*R*, *E*_ can be interpreted as a measure of each department’s precision concerning the estimation of future cash flows (henceforth referred to as general forecasting ability). If *p*_*R*, *E*_ = 1, there is no uncertainty about future cash flows and any two simulated trajectories of the bivariate GBM will perfectly coincide. However, as soon as *p*_*R*, *E*_ < 1, (i) the departments’ forecasts of cash flow paths will be more volatile, i.e., the magnitude of the change in cash flow levels will be overestimated (*σ* ^(*E*)^ > *σ*), and (ii) departments will more often misjudge the signs of the change of cash flows from one period to the next. Since 0 < *p*_*R*, *E*_ ≤ 1, each department’s ability of correctly forecasting future cash flows varies from (virtually) 0% to 100%.

We generate paired trajectories of this process for *t* = 1, 2, …, *T*, serving as the real and estimated cash flow time series for department *i* operating investment opportunity *j*. All departments, then, estimate their efficiency in carrying out the project they are involved in. The efficiency parameter is drawn from a normal distribution, hence $\rho_{ij}^{\left(E\right)}∼N(f_{\rho },\sigma^{2})$, with *f*_*ρ*_ ∈ (0, 1], and feasible $\rho_{ij}^{\left(E\right)}\in (0,1]$. However, department *i* faces uncertainty concerning its efficiency in operating investment opportunities, i.e. the real efficiency is normally distributed according to $\rho_{ij}^{\left(R\right)}\sim N(\rho_{ij}^{\left(E\right)},{\left(\sigma^{\left(E\right)}\cdot \rho_{ij}^{\left(E\right)}\right)}^{2})$, and $\rho_{ij}^{\left(R\right)}\in (0,1]$. The parameter *f*_*ρ*_ can thus be interpreted as the ability of department *i* to earn the *expected fraction of *f*_*ρ*_* of the maximum achievable cash flows **x**_*ij*_.

Given the firm’s costs of capital *r*_*c*_, the PV of each intertemporal distribution of cash flows (real as well as estimated) is, thus, defined by $PV_{ij}^{\left(k\right)}({\text{x}}_{ij}^{\left(k\right)},\rho_{ij}^{\left(k\right)},r_{c})$. Moreover, given the total estimated present value of project *j*, $PV_{j}^{\left(E\right)}=\sum_{\forall i:f\left(i,j\right)=1}{PV}_{ij}^{\left(E\right)}\left({\text{x}}_{ij}^{\left(E\right)},\rho_{ij}^{\left(E\right)},r_{}\right)$, the estimated initial cash outlay is drawn from a normal distribution, hence $\kappa_{j}^{\left(E\right)}\sim N(f_{\kappa }\cdot PV_{j}^{\left(E\right)},{\left(\sigma^{\left(R\right)}\cdot PV_{j}^{\left(E\right)}\right)}^{2})$, with *f*_*κ*_ ∈ (0, 1), and feasible $\kappa_{j}^{\left(E\right)}\in (0,PV_{j}^{\left(E\right)}]$. The latter definition guarantees positive outlays and only non-negative NPV projects as a result of the estimation process of all involved departments. Note that *f*_*κ*_ can be interpreted as the expected fraction of the initial cash outlay and the PV generated from project *j*. Further, $\frac{1-f_{\kappa }}{f_{\kappa }}$ may be seen as the expected return on investment, e.g., for *f*_*κ*_ = .8 the project will yield an expected return of $\frac{1-.8}{.8}=25\%$. The actual initial cash outlay is then drawn from normal distribution, thus $\kappa_{j}^{\left(R\right)}\sim N(\kappa_{j}^{\left(E\right)},{\left(\sigma^{\left(E\right)}\cdot \kappa_{j}^{\left(E\right)}\right)}^{2})$, and $\kappa_{j}^{\left(R\right)}>0$. Note that since the initial cash outlay necessary to launch investment opportunities is set in such a way that all projects have a positive NPV, in some cases any combination of two or more projects other than the best may result in a higher total NPV as well as a lower total outlay. We skip these simulations and repeat this particular simulation run in order to ensure that the coordinating unit’s optimal strategy is to fund only one project at most. Please also note that we differ from [10] with respect to the process for generating the initial cash outlay. [10] describe the initial cash outlay to be normally distributed with fixed boundaries, while we make the interval contingent on the respective project’s present value. Finally, real and expected NPVs are calculated for all projects, thus $NPV_{j}^{\left(k\right)}=PV_{j}^{\left(k\right)}-\kappa_{j}^{\left(k\right)}$ (see also Equation 3).

### Error measures

Basically, due to the nature of the simulation setup outlined in the previous section, the firm runs the risk of erroneously choosing projects other than the highest NPV project under *r*_*c*_. This is accounted for by incorrect departmental forecasts of intertemporal distributions of cash flows, efficiency parameters and initial cash outlays necessary to launch investment opportunities. Motivated by the corporate goal of SHV maximization, monetary effects of such a wrong decision are captured by three measures:

- Total loss (TL)
- Project loss (PL)
- Compensation loss (CL)

Recall from Equation 12 that the firm’s utility is determined by the realized NPV from project *j* minus the compensation paid to the involved departments. The compensation *π*_*ijt*_ of department *i* operating investment opportunity *j* at time *t* is a function of the residual income, hence $\pi_{ijt}=f\left(v_{ijt}\right)=f\left(x_{ijt}\cdot (\rho_{ij}-\rho_{ij}^{*})\right)$. Since $\rho_{ij}^{*}=(\Lambda_{j}^{*}+\kappa_{j})/(\Lambda_{j}+\kappa_{j})\cdot \rho_{ij}$ we may rewrite the residual income as

$$\begin{array}{c} v_{ijt}=x_{ijt}\cdot \rho_{ij}\cdot \left(1-\left(\Lambda_{j}^{✱}+\kappa_{j}\right)/\left(\Lambda_{j}+\kappa_{j}\right)\right) \end{array}$$ (14)

Given that the compensation is a linear function of the residual income, department i’s discounted total compensation from project *j* utilizing the firm’s cost of capital *r*_*c*_ is given by

$$\begin{array}{c} \Pi_{ij}=f\left(PV_{ij}\cdot (1-\left(\Lambda_{j}^{✱}+\kappa_{j}\right)/\left(\Lambda_{j}+\kappa_{j}\right)\right) \end{array}$$ (15)

Note that departments are paid in each period according to the cash flows effectively realized in the respective period. These cash flows will also most probably deviate from estimated values. For simplicity, and equivalency, however, the present value is under consideration here. The firm’s income is given by

$$\begin{array}{c} \Lambda_{j}-\sum_{\forall i:f\;\left(i,j\right)=1}\Pi_{ij}=\Lambda_{j}-f\left(\sum_{\forall i:f\left(i,j\right)=1}PV_{ij}\cdot \left(1-\left(\Lambda_{j}^{✱}+\kappa_{j}\right)/\left(\Lambda_{j}+\kappa_{j}\right)\right)\right) \end{array}$$ (16)

Now assume that the compensation for the departments takes the functional form *f*(.) = *a* ⋅ (.), where *a* is used to control the fraction of the total NPV generated by investment opportunity *j* assigned as compensation payments to the departments. The firm’s income then

$$\Lambda_{j}-a\cdot \left(\sum_{\forall i:f\left(i,j\right)=1}PV_{ij}\cdot (1-(\Lambda_{j}^{*}+\kappa_{j})/(\underset{PV_{j}}{\underset{︸}{\Lambda_{j}+\kappa_{j}}}))\right)=\Lambda_{j}-a\cdot \left(PV_{j}+\kappa_{j}-\Lambda_{j}^{*}\right)$$ (17)

Without loss of generality, we set *a* = 1, thus the firm’s income is simply $\Lambda_{j}-PV_{j}-\kappa_{j}+\Lambda_{j}^{*}=\Lambda_{j}^{*}$ and thus the departments receive a total compensation of $\Lambda_{j}-\Lambda_{j}^{*}$. It can readily be seen that the firm’s income is the NPV of the investment opportunity ranked second, whereby the remainder of the best project’s NPV is shared between the involved departments.

However, in a world without perfect foresight, the firm realizes the very investment opportunity that only *appears* to be best based on estimated values (henceforth tagged with a tilde) and further determines compensation payments based on estimated values. The NPV actually realized is given by ${\tilde{\Lambda }}^{\left(R\right)}$, whereby $\tilde{\Lambda }$ denotes the NPV that was estimated for the realized project and Λ is the NPV of the actually best project. We use the same notational conventions for the PV and the outlay *κ*. For convenience, we drop subscripts from now onwards.

Even though the firm’s periodical compensation payments are determined by realized cash flows and also accrue periodically, we assume that already at the beginning of the project the firm and the departments agree on the share of the (actually realized) cash flows taken by the firm and the departments, respectively. Note that we explicitly rule out renegotiation of compensation payments. This may be reasoned by the assumption that departments do not bear the risk of erroneous forecasts of project data, but the coordinating unit does.

Thus, the actual compensation payment $\tilde{C}$ determined by estimated values is given by

$$\begin{array}{c} \tilde{C}={\tilde{PV}}^{\left(R\right)}\cdot \left(1-\frac{{\tilde{\Lambda }}^{✱}+\tilde{\kappa }}{\tilde{\Lambda }+\tilde{\kappa }}\right) \end{array}$$ (18)

The TL from the firm’s point of view caused by the choice of an erroneous project is then

$$\begin{array}{c} \text{TL}=\underset{\text{income}\;\text{under}\;\text{perfect}\;\text{foresight}}{\underset{︸}{\Lambda -(\Lambda -\Lambda^{✱})}}-\underset{\text{realized}\;\text{income}}{\underset{︸}{\left({\tilde{\Lambda }}^{\left(R\right)}-\tilde{C}\right)}} \end{array}$$ (19)

Rearranging Equation 19 yields the definition of the PL and the CL respectively

$$\begin{array}{c} \text{TL}=\underset{\text{project}\;\text{loss}}{\underset{︸}{\Lambda -{\tilde{\Lambda }}^{\left(R\right)}}}+\underset{\text{compensation}\;\text{loss}}{\underset{︸}{\tilde{C}-\left(\Lambda -\Lambda^{✱}\right)}} \end{array}$$ (20)

The TL, thus, is comprised of the NPV that has not been realized due to the choice of the wrong project and the over- or under-compensation that departments receive based on estimated values. Note that the PL is a positive number if a wrong project is chosen, and zero if—even in the case of erroneous forecasts—the actual best investment opportunity is put into action. The CL is positive if departments receive compensation payments that are higher than in the case of operating the best project under perfect foresight. If CL is negative, the departments’ compensation is lower than in the case of operating the best project under perfect foresight.

Further, simplifying Equation 19 yields an equivalent expression of TL

$$\begin{array}{c} \text{TL}=\Lambda^{✱}-{\tilde{\Lambda }}^{\left(R\right)}+\tilde{C} \end{array}$$ (21)

Since $\tilde{C}\geq 0$, the total loss may only take negative values—which implies a *higher* income for the firm if data is estimated erroneously than with perfect foresight—if and only if the actually best project is chosen even under noisy forecasts. In other words, ${\tilde{\Lambda }}^{\left(R\right)}$ may only exceed Λ^*^ (the NPV of the investment opportunity ranked second) if the actually realized NPV of the project chosen under erroneous forecasts is that of the actually best project. As soon as the coordinating unit picks an investment project that actually yields an NPV other than the NPV of the investment opportunity ranked first, we have $\Lambda^{*}-{\tilde{\Lambda }}^{\left(R\right)}\geq 0$ and thus the firm suffers a TL ≥ 0. Since the error measures are a function of the number of departments and the number of projects, we explicitly stress that our research interest does not focus on the solution of an optimization problem (in terms of the optimal number of departments/projects). We are rather interested in deriving quantitative effects (regarding TL, PL, and CL) of erroneous forecasts.

## Simulation results

In this section, we present the results of our simulation experiments. In order to illustrate the functioning of the mechanism, we start with a brief numerical example with perfect foresight in Sect. ‘Numerical example with perfect foresight’. In Sect. ‘Preliminary analysis from erroneous forecasts’ we add uncertainty, i.e., we model departments to make errors in estimating the initial cash outlay, in their efficiency of operating the investment opportunity, and in estimating the cash flow time series earned by the respective project. In Sect. ‘Reduced number of error sources’, we, then, reduce the number of error sources, so that one or two out of the three measures associated with investment opportunities are forecasted with perfect foresight, while the remaining measures are still erroneous. This analysis is necessary to give guidance on how to sequence data quality activities. Finally, in Sect. ‘Robustness of results’ we extensively analyze the robustness of the results to variations in the main model parameters.

### Numerical example with perfect foresight

**Fig 4:**
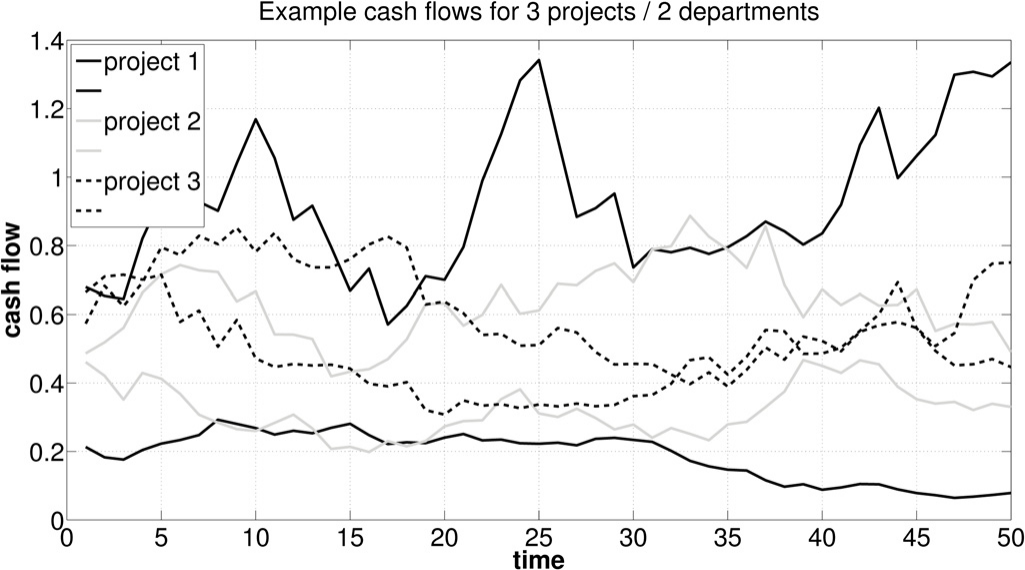
Illustration of possible intertemporal cash flow distributions in a three investment opportunities / two departments per project setup.

We model a firm, which seeks to realize one out of three projects, each operated by two departments. Hence, for the ease of understanding, in this example the number of all possible combinations of projects and departments is reduced to 2 ⋅ 3 = 6. The projects’ lifetime is fixed at 50 periods. Fig. 4 shows the intertemporal distribution of cash flows realized by each department. Under the corporate costs of capital of 10%, *PV*_*ij*_ is defined as the present value of department *i*’s cash flows from operating project *j* (see Equation 2). Thus, *PV*_11_ = 2.25, *PV*_21_ = 8.37, *PV*_12_ = 6.01, *PV*_22_ = 3.32, *PV*_13_ = 5.49, and *PV*_23_ = 6.95. We randomly generate outlays for project *j* of *κ*_1_ = 10.44, *κ*_2_ = 6.19, and *κ*_3_ = 10.11, yielding project NPVs of Λ_1_ = .17, Λ_2_ = 3.14, and Λ_3_ = 2.32 (see Equation 3).

As one can see, the firm maximizes SHV by providing incentives so that investment opportunity 2 from the alternatives available is operated by departments. In a next step, we derive critical efficiencies (see Equation 5) and the cost of capital (see Equation 6) for each investment opportunity’s aggregated departmental cash flows. As a result, the following hurdle rates for project *j* may be obtained: $r_{1}^{*}=1.5262$, $r_{2}^{*}=.1121$, and $r_{3}^{*}=.1758$. The share of the initial outlay for project *j* assigned to department *i*, *λ*_*ij*_, is then *λ*_11_ = .2290, *λ*_12_ = .6405, and *λ*_13_ = .4618. Note that the other of the two departments involved in one project is always assigned a share of 1−*λ*_1*j*_. Under the hurdle rate based coordination mechanism and the particular share of the initial cash outlay, all departments recalculate their individual NPVs, Λ_*ij*_(*r* ^*^, *λ*_*ij*_) = *PV*_*ij*_ − *λ*_*ij*_ ⋅ *κ*_*j*_, from operating project *j*. Results are as follows: Λ_11_ = −2.26, Λ_21_ = −7.60, Λ_12_ = 1.40, Λ_22_ = .79, Λ_13_ = −1.22, and Λ_23_ = −1.42. Thus, both departments involved in investment opportunity 2 will independently opt for operating their joint project, while departments involved in project 1 or 3 will refrain from realizing their joint project.

### Preliminary analysis from erroneous forecasts

This section adds uncertainty into the simulation design and, in detail, examines the effects of imperfect departmental forecasts concerning measures associated with investment opportunities, i.e., the intertemporal distribution of cash flows, the initial cash outlay necessary to launch investment opportunities, and the departments’ efficiency in operating projects. All relevant parameters utilized during our simulation runs, either fixed or subject to variation, are presented in Table 2.

**Table 2:** Parameters in the simulation setup.

Parameter	Description	Range
*μ*_*ij*_	drift parameter of the cash flow process	fixed, 0
*σ*	general risk parameter	fixed, .1
*p*_*R*, *E*_	correlation of real and estimated cash flow trajectories	variable, {.2, .4, .6, .8}, equal for all departments
*T*	number of periods of the project	fixed, 5
*r*_*c*_	corporate cost of capital	fixed, .1
$\rho_{ij}^{\left(E\right)}$	estimated efficiency parameter of department *i* operating project *j*	random, $\rho_{ij}^{\left(E\right)}∼\text{N}(f_{\rho },\sigma^{2})$, with *f*_*ρ*_ = .5, $\rho_{ij}^{\left(R\right)}\sim N(\rho_{ij}^{\left(E\right)},{\left(\sigma /p_{R,E}\right)}^{2})$
$\kappa_{j}^{\left(E\right)}$	estimated initial outlay of project *j*	random, $\kappa_{ij}^{\left(E\right)}\sim N(f_{\kappa }PV_{j}^{\left(E\right)},{\left(\sigma \cdot PV_{j}^{\left(E\right)}\right)}^{2})$, with *f*_*κ*_ = .5, $\kappa_{ij}^{\left(R\right)}\sim N(\kappa_{ij}^{\left(E\right)},{\left(\sigma /p_{R,E}\cdot \kappa_{ij}^{\left(E\right)}\right)}^{2})$
*n*	number of projects	variable, {2, 3, 4, 5, 6, 7, 8}
*z*	number of departments operating one project jointly	variable, {2, 3, 4, 5, 6, 7, 8}
*s*	number of simulation runs	fixed, 10,000

We start by analyzing the effects of different structures underlying the coordination mechanism, i.e. the effect of different numbers of projects and departments operating these projects, respectively. The number of departments, which take part in one round of budget allocation can, to some extent, be regarded as one of the organization’s policy parameters as it can easily be stipulated by the coordinating unit (or a higher hierarchical level) as a precondition for the application for funding (see also [10]). The same applies to the number of applications, which are considered in one round of budget allocation. Thus, it appears that the number of projects and departments operating these projects are feasible policy parameters for the context of our investigation.

**Fig 5:**
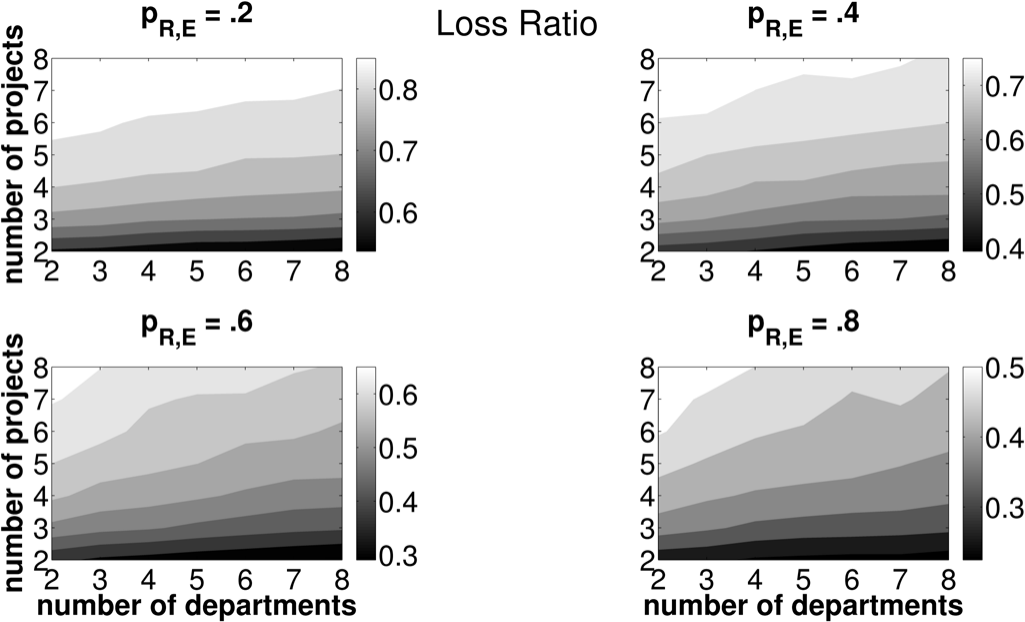
Loss ratio (LR) for different combinations of *n*, *z* and *p*_*R*, *E*_. Light (dark) areas indicate a high (low) LR. Individual subplots refer to varying forecasting abilities *p*_*R*, *E*_.

Fig. 5 reports results from 10,000 simulation runs concerning the ratio of sub-optimally operated investment opportunities, i.e., cases where projects other than the one ranked first (in terms of NPV) are selected to be operated. The corresponding measure is called loss ratio (LR). Light (dark) areas in each subplot indicate high (low) LR, subplots refer to values of *p*_*R*, *E*_ of {.2, .4, .6, .8} respectively. The abscissa (ordinate) shows varying numbers of departments (projects). It turns out that LR primarily increases with the number of projects and slightly decreases with the number of departments operating one joint project. In other words, companies that decide to consider a larger number of investment opportunities proposed by two or more departments, respectively, in one round of budget allocation, face a higher probability of choosing the wrong project due to inaccurate forecasts of the intertemporal distribution of cash flows and the departmental efficiency in operating projects. Furthermore, they are more likely to erroneously assess the necessary cash outlay in order to operate the project. This pattern persists for all levels of forecasting accuracy, *p*_*R*, *E*_, even though the absolute level of LR is—not surprisingly—decreasing if forecasting accuracy increases. Please recall that a higher value of *p*_*R*, *E*_ corresponds to a higher accuracy in forecasting. If the firm seeks to minimize the probability of selecting an adverse investment opportunity, a low number of available projects to be considered in one round of budget allocation should be targeted.

**Fig 6:**
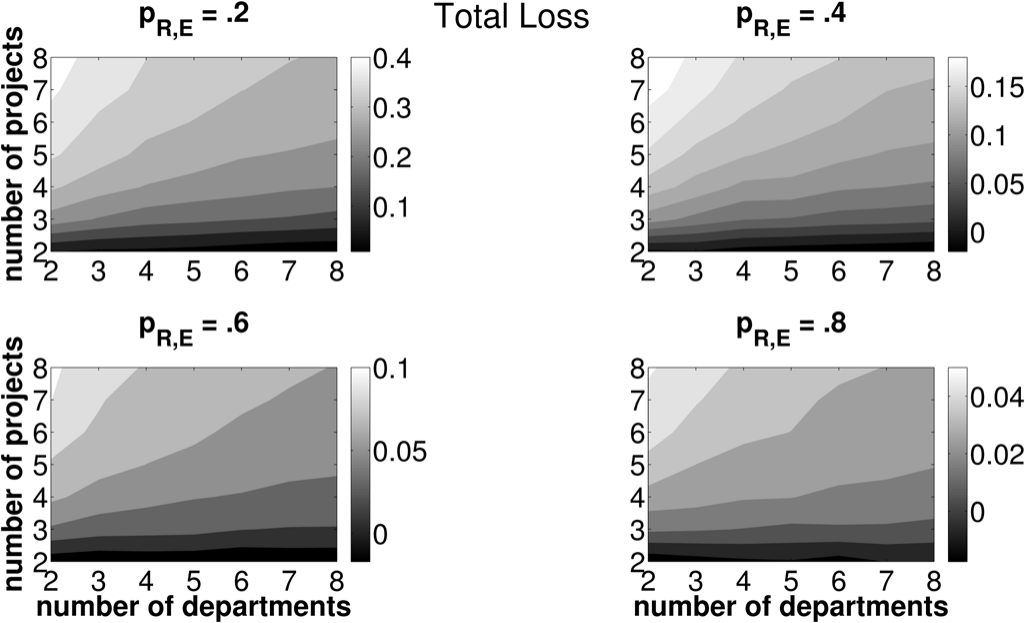
Mean TL for different combinations of *n*, *z* and *p*_*R*, *E*_. Light (dark) areas indicate a high (low) TL. Individual subplots refer to varying forecasting abilities *p*_*R*, *E*_.

**Fig 7:**
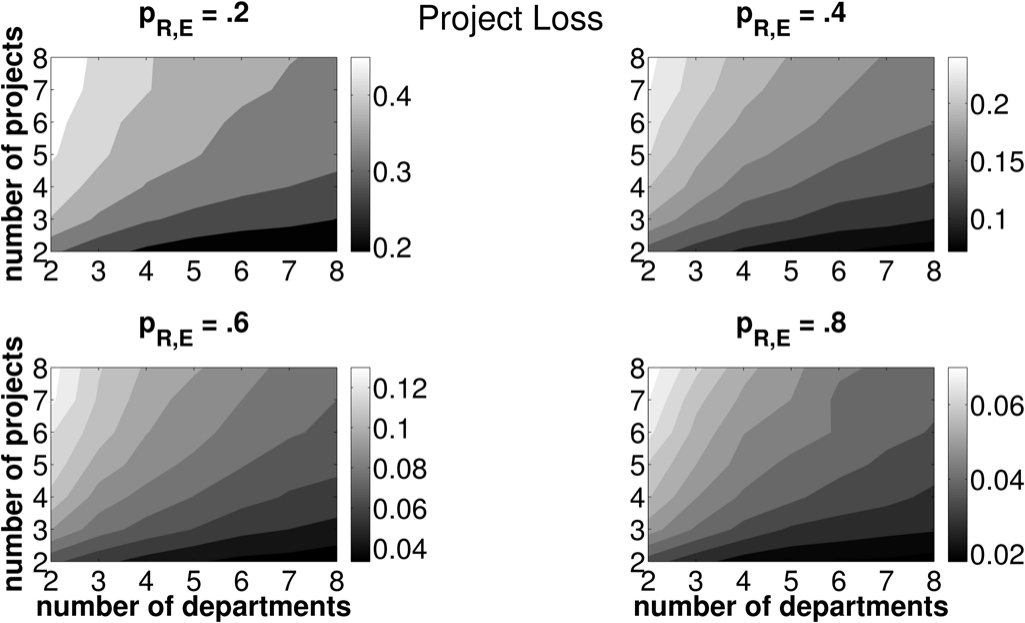
Mean PL for different combinations of *n*, *z* and *p*_*R*, *E*_. Light (dark) areas indicate a high (low) PL. Individual subplots refer to varying forecasting abilities *p*_*R*, *E*_.

**Fig 8:**
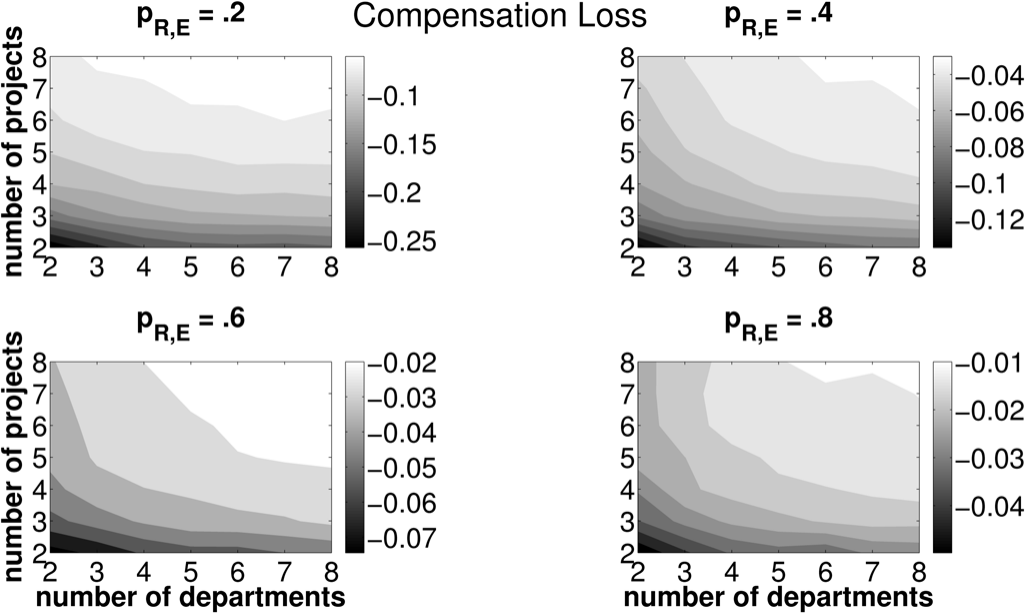
Mean CL for different combinations of *n*, *z* and *p*_*R*, *E*_. Light (dark) areas indicate a high (low) CL. Individual subplots refer to varying forecasting abilities *p*_*R*, *E*_.

However, LR itself does not necessarily indicate desirable and non desirable framework conditions for an efficient functioning of the allocation mechanism (in terms of the number of investment opportunities and departments operating them). In a next step, we therefore investigate the effect of varying numbers of projects and departments on the error measures TL, PL, and CL. Due to the nature of the simulation setup, a higher number of departments operating one joint investment project implies both a higher total expected PV and NPV. This is due to the fact that all departmental cash flow distributions have the same expected PV and doubling the number of departments is equivalent to doubling the expected project PV. We, therefore, normalize all error measures by the present value of the best project based on estimated data, $\tilde{PV}$. Figs. 6–8 report results on the effects of varying numbers of departments and projects on these error measures. As Fig. 6 shows, TL increases with the number of available investment opportunities and decreases with the number of departments operating the investment projects. This pattern is similar to that observed with LR, yet, the decrease in TL with an increasing number of departments is more pronounced than with LR. Put differently, picture a firm with five projects and five departments jointly operating one project,, respectively. Ceteris paribus,

- reducing (increasing) the number of departments operating one joint project (i.e., moving to the left (right) in the plot) increases (decreases) TL
- reducing (increasing) the number of projects, which are considered in one round of budget allocation (i.e., moving downward (upward) in the plot) decreases (increases) TL

Hence we can preliminarily conclude, if the firm seeks to minimize TL due to inaccurate forecasting activities and, as a consequence, the selection of adverse projects, the framework conditions for the coordination mechanism should feature a low number of available investment opportunities, each operated by a large number of conjoint departments. Furthermore, for *p*_*R*, *E*_ ≥.4, we actually observe negative values for TL in any setup featuring two projects only. Irrespective of the number of departments jointly operating one out of two projects, the firm will have an even higher increase in SHV as compared to error-free forecasts. From this point of view, any firm selecting from only two investment opportunities will prefer errors in project related forecasts over perfect foresight! For high forecasting abilities and a sparse two-projects-setup, it is likely that despite erroneous forecasting activities the actual best investment opportunity will be operated and, thus, the PL is zero. The firm and the departments involved in the ‘winning’ project agree on compensation payments based on estimated values. Due to erroneous forecasting, the fraction of the realized cash flows assigned to the departments may be lower than without errors. In fact, the simulation results show that, on average, too few compensation payments are put into effect in all scenarios where only two investment opportunities are considered in one round of budget allocation (i.e, the bottom-left corner of the contour plots in Fig. 6). From this point of view we can conclude that, acting on SHV maximization, the firm should restrict the pool of available projects for one round of budget allocation to two and strive for high—yet not perfect—forecasting abilities on the part of its departments.

Fig. 7 and 8 disentangle the TL measure and depict the contribution of PL and CL to the overall measure TL. PL, which is depicted in Fig. 7, features the same properties as TL (see Fig. 6) with regard to different framework conditions for the coordination mechanism, in terms of the number of departments and projects. By definition, PL is strictly positive and further features an increase with the number of projects and a decrease with the number of departments. Interestingly, the mean CL is negative—we may therefore call it a compensation *gain*—in all combinations of investment opportunities, *n*, the number of departments, which are involved in one project, *z*, and forecasting abilities, *p*_*R*, *E*_ (see Fig. 8). In other words, the firm will always, on average, under-compensate the departments for operating their joint project irrespective of the number of projects and the number of departments as well as of general forecasting abilities. The compensation gain is particularly high for firms featuring a low number of investment opportunities in each round of budget allocation and each of these opportunities is jointly operated by only few departments.

With respect to TL, we thus have two opposing effects: On the one hand, the firm may suffer from a PL, if, due to inaccurate forecasts, a project other than the project ranked first in terms of NPV is operated. On the other hand, the compensation payments assigned to the departments based on estimated project data, on average fall below the level of compensation payments based on data under certainty. Therefore, PL is consistently mitigated by a negative CL (which is, from the firm’s perspective, in fact a ‘compensation gain’). However, as outlined in Equation 21, the compensation gain may never exceed the loss in NPV from choosing an adverse project. Recall the definition of the total loss: $\text{TL}=\Lambda^{*}-{\tilde{\Lambda }}^{\left(R\right)}+\tilde{C}$. A negative TL may only occur in cases where the actually best project is chosen by the firm. As soon as erroneous forecasts make the firm pick an adverse investment opportunity, PL is strictly positive and TL cannot be negative. Hence, for scenarios with adverse projects being chosen, PL must always exceed the compensation gain in order to satisfy the condition of a positive TL.

### Reduced number of error sources

This section outlines effects of a reduced number of erroneously forecasted measures associated with investment projects. Recall that, in the basic setup, the intertemporal distribution of cash flows, **x**, the initial cash outlays necessary to launch a project, *κ*, and the departments’ efficiencies in operating investment opportunities, *ρ*, are subject to misforecasting. In this section, we provide results in scenarios in which uncertainty for one or two out of these three error sources is dropped. In other words, either one or two values from the set {*x*, *κ*, *ρ*} are known at the very beginning of the forecasting process. We use the same parameter sets as in Sect. ‘Preliminary analysis from erroneous forecasts’ and conduct an analysis concerning TL, PL and CL respectively for the setups displayed in Table 3. Basically, the patterns from the baseline scenario concerning TL, PL and CL remain unchanged in a world with knowledge on one or two measures associated with investment projects in the departments’ forecasting process. That is, when departments face fewer error sources, TL as well as PL are generally low for any number of departments jointly operating one out of only two available investment opportunities. Further, CL is lowest for a two departments, two projects setting (henceforth denoted by 2/2). In this regard, the results presented in this section do not differ qualitatively from those in the preliminary results Sect. ‘Preliminary analysis from erroneous forecasts’. However, we are able to clearly document that—if one or two parameters are forecasted without error—the extent of the *change* in the loss measures crucially depends on the framework conditions under which the coordination mechanism is deployed. This fact gives rise to a twofold examination of the effects of a reduced number of error sources.

**Table 3:** Overview of known and unknown parameters in alternative (reduced error) simulation scenarios.

setup	baseline	1	2	3	4	5	6
known	-	{*x*}	{*κ*}	{*ρ*}	{*x*, *κ*}	{*x*, *ρ*}	{*κ*, *ρ*}
unknown	{*x*, *κ*, *ρ*}	{*κ*, *ρ*}	{*x*, *ρ*}	{*x*, *κ*}	{*ρ*}	{*κ*}	{*x*}

First, depending on which parameters in the estimation process can (cannot) be perfectly forecasted, we are able to specify the particular framework conditions in terms of the number of investment opportunities and departments operating them, that minimize the loss measures. This is of particular interest for firms which can alter the policy parameters (i.e., the number of projects and departments per project) in the short-term. As soon as departments perfectly forecast one out of three relevant parameters it may be necessary to adapt the framework conditions in order to minimize the TL measure. Provided that the firm features this flexibility it will always be able to install framework conditions that minimize losses.

Second, if a firm lacks flexibility in adopting its framework conditions for the coordination mechanism but instead has to stay with the current number of departments and projects, learning to perfectly forecast one specific hitherto unknown parameter might have contrasting effects depending on the prevailing framework conditions. In other words, perfect knowledge on, e.g., the initial cash outlay may decrease TL for a firm featuring a high number of projects and departments, but, by contrast, may even increase TL, if the firm selects from a low number of projects jointly operated by only few departments.

Further, since gaining better forecasting abilities most likely entails cost (such as research or information cost), a firm learning to better forecast project related measures is required to focus on better forecasting the very parameter(s) inducing the maximum decrease in the relevant loss measures.

We want to start with displaying the number of departments and projects, exhibiting the lowest (highest) absolute levels of TL, PL and CL respectively. Our simulation results based on a reduced number of error sources indicate that along with results from the preliminary analysis, loss measures are minimized (maximized), if the number of investment opportunities considered in one round of funding is low (high). In particular, the minimum of all three loss measure (TL, PL and CL) is associated to only two available projects in all simulation setups under consideration. Contrary, loss measures almost always hit their maximum if the number of available investment opportunities is set to eight. Hence, Tables 4 and 5 only report the number of departments connected to minimizing (maximizing) loss measures. As Table 4 points out, depending on general forecasting abilities (*p*_*R*, *E*_ = {.2, .8}) and on which parameters from the set {*x*, *κ*, *ρ*} can (cannot) be perfectly forecasted, the number of departments minimizing loss measures is widely different. Additional knowledge on one or two parameters possibly requires the firm to adapt the number of departments considered in the current round of budget allocation in order to hit the framework conditions minimizing TL. For flexible firms, adapting our proposed policy parameters should be able to be undertaken quite easily. However, firms without this flexibility may only want to gain knowledge on these parameters, which do not imply the necessity of changing the number of departments. Hence, starting from the baseline setup with unknown {*x*, *κ*, *ρ*}, firms with departments having low (high) forecasting abilities will strive after learning to perfectly forecast parameters. This does not call for a change in the framework conditions. Besides a general presentation of the minima and maxima of the three loss measures dependent on the number of known parameters as well as general forecasting abilities of the departments, Table 6 highlights the results to which it comes down effectively. The firm seeks to minimize TL, which represents the missed NPV and the differences in the amount of the departments’ compensation, which are due to erroneous forecasts and as a consequence the choice of an adverse project. In fact, the departments’ compensation depends on the PV generated from the operated project, but it also crucially depends on the gap between the ‘winning project’s’ NPV and the NPV of the project ranked second from the remaining set of applications for funding (cf. also [23]). Erroneous forecasts thus enable the departments to be overcompensated as compared to error free project appraisals—irrespective of whether the actual best or an adverse project is finally carried out. From this point of view, departments are not primarily interested in the correct choice of the SHV maximizing project, but rather in maximizing their compensation payments. Correspondingly, Table 6 finally contrasts the respective framework conditions for deploying the proposed coordination and budget allocation mechanism showing the minimum TL as well as the maximum CL for bad (.2) and good (.8) general forecasting abilities. The motivation behind this can be found in the firm’s attempt to minimize TL (equivalent to maximizing the SHV) and the departments’ aim to maximize CL (equivalent to being overcompensated / less undercompensated). Table 6 clearly points out that the firm’s choice of the number of departments and investment opportunities considered in one round of funding in order to minimize TL is mostly the exact opposite of the framework conditions that departments would choose to maximize their own compensation, which corresponds to maximizing CL (e.g., if {*x*} is known, the firm strives for a 2/2 structure, whereas the departments prefer an 8/8 structure). We therefore might conclude that the firm and the departments pursue conflicting goals concerning the framework conditions. However, this is only true, if we keep with the relative definition of the CL measure. Recall that CL is related to the estimated best project’s PV. Hence, the measure captures a percentage of the departments’ undercompensation. Nevertheless, the *absolute values* of average compensation payments per department always reach their maximum in a 2/2 setup (for bad as well as good general forecasting abilities). Thus, the departments’ preferences for the framework conditions partly correspond to the firm’s preferences (in particular if {*x*} or {*x*, *κ*} are known or for the case of *p*_*R*, *E*_ = .8 and known {*κ*}).

**Table 4:** Reduced error simulation scenarios (minimized loss measures).

known	-	{*x*}	{*ρ*}	{*κ*}	{*x*, *ρ*}	{*x*, *κ*}	{*κ*, *ρ*}
unknown	{*x*, *κ*, *ρ*}	{*κ*, *ρ*}	{*x*, *κ*}	{*x*, *ρ*}	{*κ*}	{*ρ*}	{*x*}
min TL (.2)	8	2	8	8	8	2	8
min TL (.8)	3	2	8	2	5	2	8
min PL (.2)	8	7	8	8	2	8	8
min PL (.8)	8	8	8	8	2	8	8
min CL (.2)	2	2	8	2	8	2	2
min CL (.8)	2	2	4	2	5	2	2

The entries indicate the number of departments, which are involved in the budget allocation process that minimizes loss measures (TL, PL, CL) for bad (.2) and good (.8) general forecasting abilities. Columns show results for different combinations of known and unknown parameters in the estimation process.

**Table 5:** Reduced error simulation scenarios (maximized loss measures).

known	-	{*x*}	{*ρ*}	{*κ*}	{*x*, *ρ*}	{*x*, *κ*}	{*κ*, *ρ*}
unknown	{*x*, *κ*, *ρ*}	{*κ*, *ρ*}	{*x*, *κ*}	{*x*, *ρ*}	{*κ*}	{*ρ*}	{*x*}
max TL (.2)	2	2	2	2	8	2	2
max TL (.8)	2	2	2	2	6	2	2
max PL (.2)	2	2	2	2	8	2	2
max PL (.8)	2	2	2	2	5	2	2
max CL (.2)	5	8	2	8	5	8	6
max CL (.8)	8	8	8	8	8	8	4

The entries indicate the number of departments, which are involved in the budget allocation process that maximizes loss measures (TL, PL, CL) for bad (.2) and good (.8) general forecasting abilities. Columns show results for different combinations of known and unknown parameters in the estimation process.

**Table 6:** Reduced error simulation scenarios (departments/projects).

known	-	{*x*}	{*ρ*}	{*κ*}	{*x*, *ρ*}	{*x*, *κ*}	{*κ*, *ρ*}
unknown	{*x*, *κ*, *ρ*}	{*κ*, *ρ*}	{*x*, *κ*}	{*x*, *ρ*}	{*κ*}	{*ρ*}	{*x*}
min TL (.2)	8/2	2/2	8/2	8/2	8/2	2/2	8/2
max CL (.2)	5/8	8/8	2/8	8/8	5/8	8/8	6/8
min TL (.8)	3/2	2/2	8/2	2/2	5/2	2/2	8/2
max CL (.8)	8/8	8/8	8/8	8/8	8/8	8/8	4/8

The pairs of numbers (departments/projects) indicate the corporate structure to minimize (maximize) the total loss (compensation loss).

From the firm’s as well as from the departments’ point of view these results clearly document the preference for a low number of available projects, on the one hand. On the other hand, depending on the number of parameters subject to misestimation, the firm tends towards either a high or a low number of departments jointly operating one out of the two investment opportunities at choice. Contrary, if the departments strive after a maximum *absolute compensation*, their choice will always be a 2/2 setup. Further, if the departments seek to maximize CL, in general a large number of projects is targeted. These results indicate conflicting interests concerning the corporate structure, which are presumably often resolved by the firm’s autonomous decision concerning the number of departments and investment opportunities altogether.

**Fig 9:**
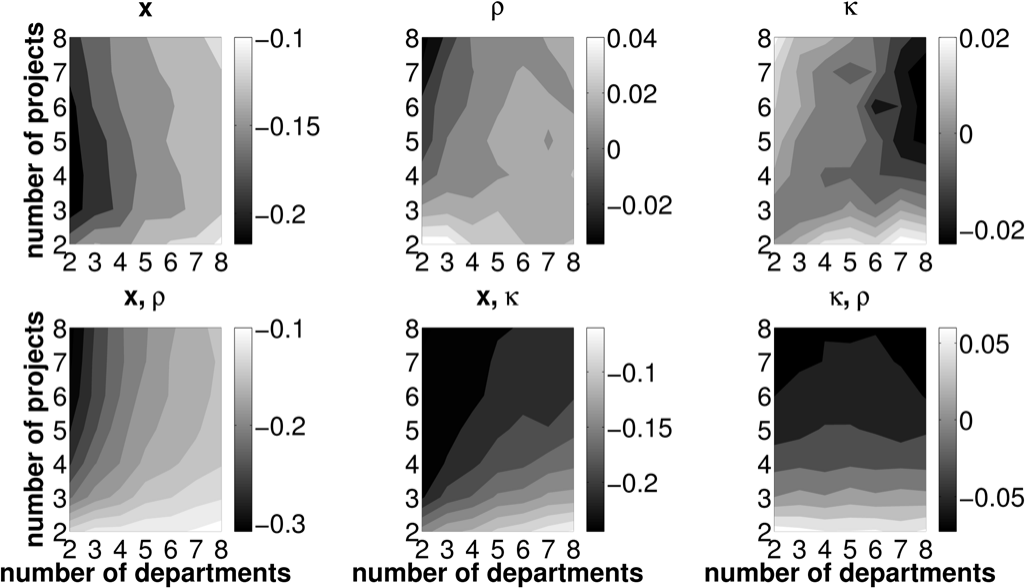
Differences in TL for *p*_*R*, *E*_ = .2. The subplots’ titles refer to the known parameter(s).

**Fig 10:**
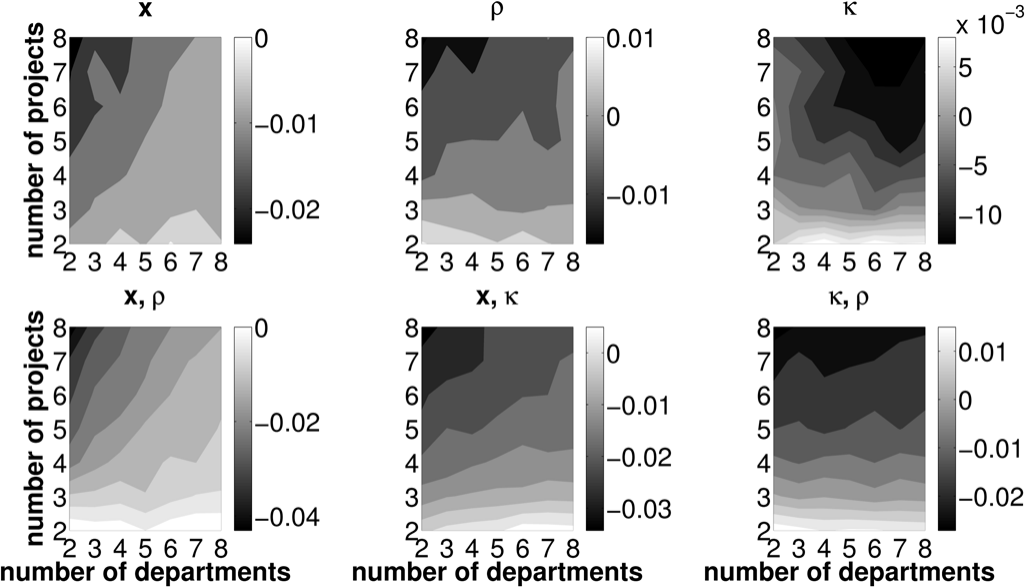
Differences in TL for *p*_*R*, *E*_ = .8. The subplots’ titles refer to the known parameter(s).

**Fig 11:**
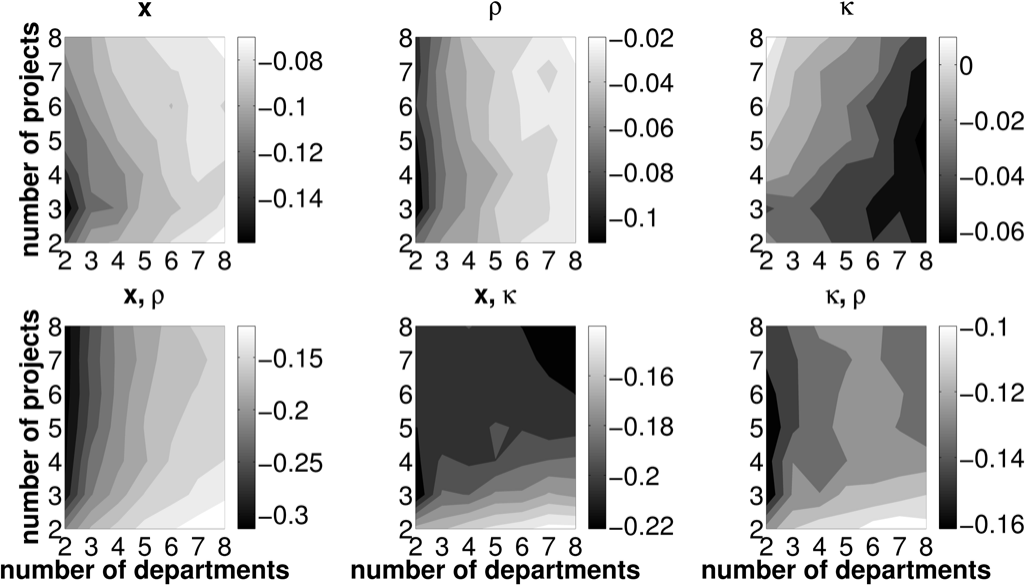
Differences in PL for *p*_*R*, *E*_ = .2. The subplots’ titles refer to the known parameter(s).

**Fig 12:**
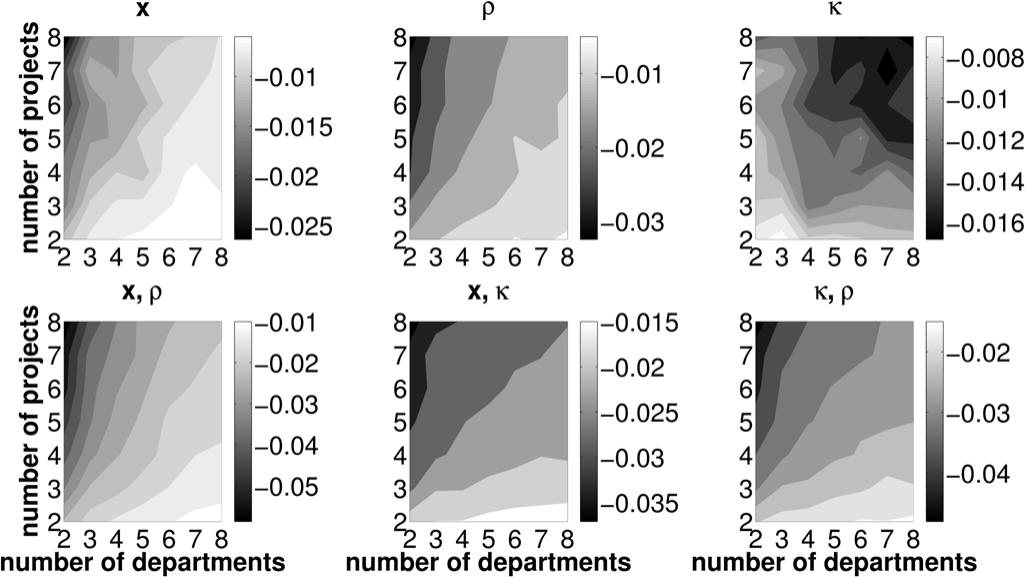
Differences in PL for *p*_*R*, *E*_ = .8. The subplots’ titles refer to the known parameter(s).

**Fig 13:**
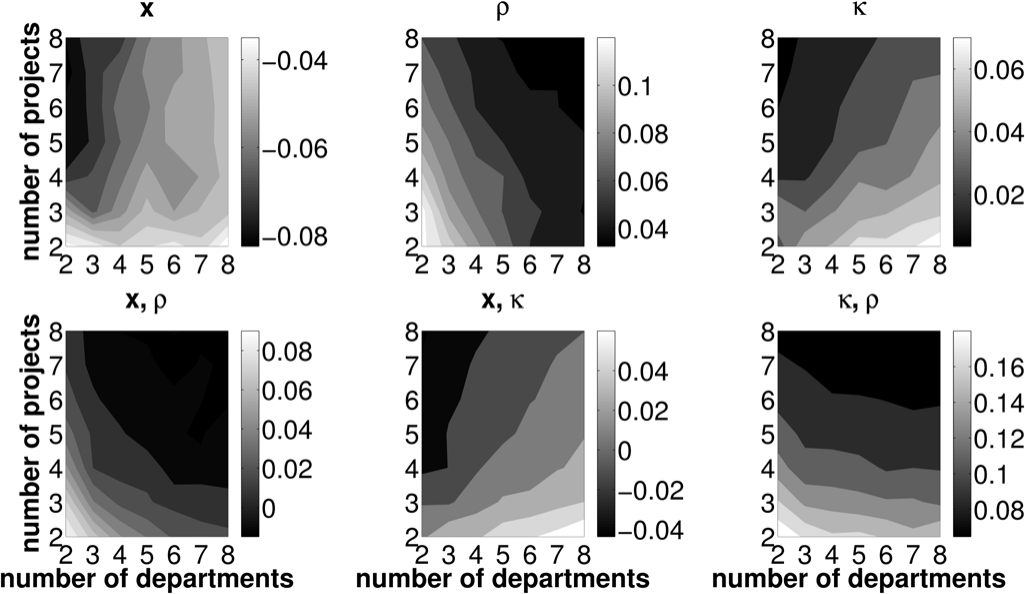
Differences in CL for *p*_*R*, *E*_ = .2. The subplots’ titles refer to the known parameter(s).

**Fig 14:**
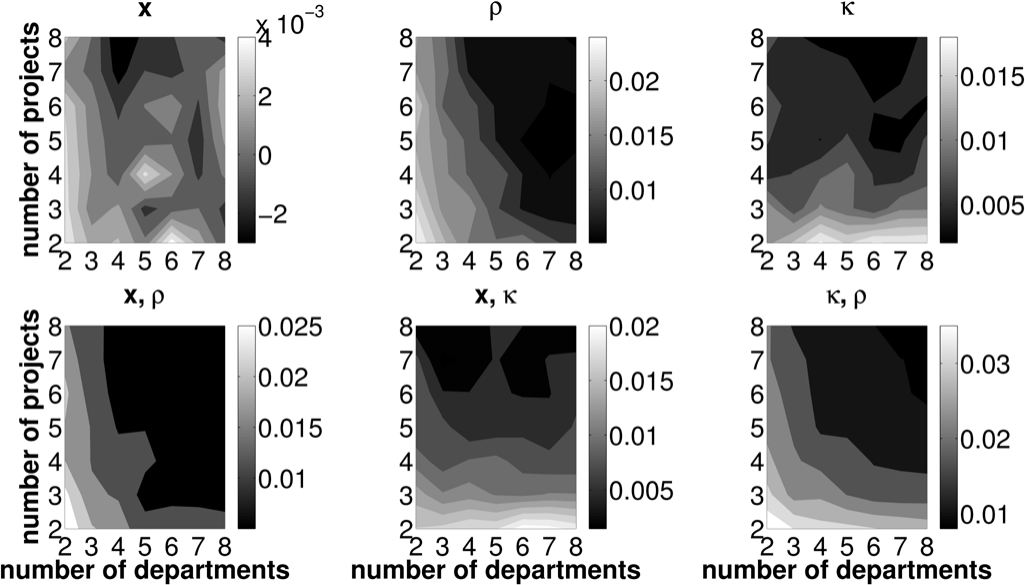
Differences in CL for *p*_*R*, *E*_ = .8. The subplots’ titles refer to the known parameter(s).

Next, we report results on the change for loss measures if one or two hitherto unknown parameters in the estimation process are fully predictable. For the sake of simplicity, we report all results graphically in terms of changes in error measures as compared to the standard setup where {*x*, *κ*, *ρ*} are subject to errors altogether. Basically, all subsequent figures’ subplots report the change in TL, PL and CL, if one or two parameters of the set {*x*, *κ*, *ρ*} are known in advance. According to the Figs. in Sect. ‘Preliminary analysis from erroneous forecasts’ this is done for different combinations of the number of investment opportunities *n* and the number of departments *z* operating one joint project. We further only report results on bad and good general forecasting abilities (hence, *p* = {.2, .8}). From the firm’s point of view negative (positive) values in Figs. 9–14 imply decreasing (increasing) levels of the respective error measure and thus perfect knowledge on either one or two parameters in the estimation process brings about beneficial (unfavorable) effects. Counter-intuitively, removing uncertainty for one or two error sources does not generally reduce losses. This may be reasoned with the fact that a higher number of error sources features smoothing effects and lower levels of losses likewise. Since all error measures may be interpreted as losses in terms of a percentage of the estimated best project’s PV, changes in all three error measures can also easily be interpreted and compared. Figs. 9 and 10 show effects in TL for bad and good forecasting abilities. The knowledge of {**x**}, {**x**, *ρ*} and {**x**, *κ*} generally lowers TL, whereas perfect foresight of {*ρ*}, {*κ*} and {*κ*, *ρ*} affects the level of the total loss differently depending on the particular number of departments and investment opportunities available. Hence, knowledge of either one or two parameters may have both positive and negative effects. For instance, perfect knowledge of the initial cash outlay increases (decreases) TL for a low (high) number of available projects whereas the knowledge of the departments’ efficiency in operating investment opportunities is particularly beneficial if the firm selects from a high number of projects jointly operated by a low number of departments. Figs. 11 and 12 depict the reduction of PL for different combinations in the number of departments and investment opportunities dependent on which parameter(s) can be perfectly forecasted by departments. Basically, PL is reduced in all scenarios. We observe PL to be significantly reduced if only few departments jointly operate one project (see left-hand side of subplots). Interestingly, this is not true if *κ* can be perfectly forecasted. In this scenario, PL can be reduced in particular for corporate structures featuring a high number of departments and a high number of available projects. As Figs. 13 and 14 point out, perfect knowledge on one or two values in the departments’ estimation process does not necessarily reduce errors in terms of lower CL. Actually, the opposite is true. Except for the scenario with perfect knowledge of **x**, CL is higher than in the case of the baseline scenario without knowledge of any of the three parameters of the set {*x*, *κ*, *ρ*}. Therefore, by analogy with results from the preliminary analysis, two opposing effects may be documented. In reduced error scenarios, knowledge of one or two parameters reduces PL for almost all combinations of the number of departments, investment opportunities and general forecasting abilities. However, CL is increased in the majority of cases, thus, the effect of reduced errors on TL largely depends on the particular structure of the firm (in terms of the number of departments and projects, and general forecasting abilities).

### Robustness of results

The simulation setup presented in Sect. ‘Simulation Setup’ makes use of parameters, which have not been subject to variation so far. Here we relax the assumption of fixed parameters depicted in Table 2, and perform a ceteris paribus analysis in order to examine the robustness of the results. That is, the drift and diffusion rate of the process for generating the intertemporal distribution of cash flows are varied. Furthermore, we consider different values of the time span of the investment project and the corporate cost of capital as well as the values of the parameters *f*_*κ*_ and *f*_*ρ*_ determining the expected cash outlay and the expected efficiency, respectively. Table 7 summarizes the values of parameters in the standard setup and states alternative values considered in the ceteris paribus analyses.

**Table 7:** Values of the parameters in the baseline scenario and alternative values for the ceteris paribus analyses.

parameter	baseline	decrease	increase
*T*	5	3	7
*r*_*c*_	.1	.05	.15
*σ*	.1	.05	.15
*μ*	0	-.02	.02
*f*_*κ*_	.5	.2	.8
*f*_*ρ*_	.5	.2	.8

In the previous sections, we thoroughly investigated effects of different corporate structures as well as general forecasting abilities of the departments. The corresponding parameters *n* (number of investment opportunities) and *z* (number of departments operating one investment opportunity) may be regarded as more or less freely controllable by the firm. The same applies to forecasting abilities captured by *p*_*R*, *E*_. This parameter may be influenced, e.g., by altering R&D expenses. Conversely, the parameter set {*T*, *r*_*c*_, *σ*, *μ*, *f*_*κ*_, *f*_*ρ*_} is likely to be determined by external conditions like the specific industry the firm belongs to. Consequently, it is beyond the firm’s influence.

From an economic point of view, varying *T* accounts for different industries’ typical time frames for carrying out investment projects. A modified *T* further accounts for altered expected total PVs since the number of cash flows changes with the number of periods. Thus an increased (decreased) *T* implies higher (lower) project PVs.

It is widely accepted that a firm’s cost of capital, *r*_*c*_, is a function of risk inherent to the firm. This is equally true for creditors (providing credit) as well as investors (providing equity). Therefore, different levels of *r*_*c*_ correspond to industries exposed to different levels of risk. Further, since *r*_*c*_ serves as the discount rate for departmental cash flows, each project’s PV will directly be affected by varying levels of the cost of capital. The corporate cost of capital does not only mirror the industry’s risk and, as a result, affect the absolute value of a project’s PV, but also may shift the PV/NPV-rank order of all available projects.

When it comes to the general risk parameter *σ* in the simulation setup, changing values entail a number of effects, namely effects on the variance of the intertemporal distribution of cash flows as well as the variance of the efficiency parameter and the variance of the initial cash outlay. Basically, a lower (higher) value decreases (increases) the discrepancy between estimated project data and real project data. Transferring this idea into a real world business, the particular value of *σ* captures the extent to which a firm is exposed to uncertainty concerning the financial appraisal of planned investment projects.

Further on, changing the drift parameter of the GBM process, *μ*, will assess the effect of systematically increasing (decreasing) levels of cash flows over time. For instance, mature industries, i.e. saturated markets, may generally face decreasing levels of cash flows whereas newly opened markets may rather be connected to increasing cash flows over time. Hence, from an economic point of view different values for *μ* can be interpreted as reflecting market saturation.

We already pointed out that *f*_*κ*_, more precisely (1 − *f*_*κ*_)/*f*_*κ*_ expresses a project’s return on investment. Since *f*_*κ*_ denotes a project’s expected outlay as a fraction of the PV of all associated cash flows, a lower (higher) value of this parameter implies a higher (lower) return on investment. This parameter may thus serve for calibrating the profitability of a specific industry. Presumably, *f*_*κ*_ is closely related to *μ* since low (high) values for the return on investment are likely to be linked to decreasing (increasing) levels of cash flows. From this perspective *f*_*κ*_ also reflects whether the state of a market can be described as growing or saturated.

Finally, as the parameter *f*_*ρ*_ determines the expected value of the departments’ efficiency, it may also be interpreted as the fraction of cash flows the departments are actually able to realize. Effectively, low (high) values of this parameter imply lower (higher) absolute values of project PVs and thus account for industries with different investment volumes.

Tables 8–10 show results from a ceteris paribus analysis for all three loss measures (TL, PL, CL). Since varying *r*_*c*_ and *μ* leaves results unchanged, for brevity we exclude these parameters from a detailed presentation. All Tables present changes in TL, PL and CL, respectively, related to the results of the baseline setup. Since all loss measures are defined relative to the best project’s PV, changes may easily be interpreted and compared. For instance, a decreased value of *f*_*κ*_ in a 8/2 setup with bad general forecasting abilities (*p*_*R*, *E*_ = .2 increases TL by 5 percentage points (see Table 8). Concomitantly, PL is decreased by 8 percentage points (see Table 9) and CL is increased by 13 percentage points (see Table 10), which makes up an increase in TL of 13-8 = 5. The right hand side of Table 8 points out that for good general forecasting abilities (*p*_*R*, *E*_ = .8) results from the baseline simulation setup mostly remain unchanged—meaning low absolute values of changes—even though changes significantly differ from zero. Conversely, when looking at the left hand side of Table 8, for bad general forecasting abilities (*p*_*R*, *E*_ = .2) almost all changes are different from zero and further show substantial absolute values. Basically, we observe a clear pattern: Decreasing (increasing) values of the parameters under consideration entail a decreasing (increasing) TL—irrespective of the particular framework conditions under which the coordination and budget allocation mechanism is deployed. However, we notice two exceptions from this pattern: decreasing *f*_*κ*_ only implies a decreasing TL for a large number of projects (2/8 and 8/8) whereas TL is increased for a low number of projects (2/2 and 8/2). We further observe effects induced by the number of projects with *f*_*ρ*_. PL is basically lower (higher) for lower (higher) values of the parameters in the simulation setup, both for bad and good general forecasting abilities (see Table 9). Contrary to TL and PL, CL does not generally tend to decrease (increase) with decreasing (increasing) values of parameters. Instead we observe a diverse pattern, at least for bad general forecasting abilities (left hand side of Table 9). For good general forecasting abilities (see right hand side of the Table 10) changes in CL are frequently different from zero, however, absolute changes are negligible.

**Table 8:** Changes in TL for changes in individual parameters.

		*p*_*R*, *E*_ = .2				*p*_*R*, *E*_ = .8			
	d/p	2/2	2/8	8/2	8/8	2/2	2/8	8/2	8/8
*T*	dec	-.02*	-.02*	-.02*	-.03*	.00	.00*	.00	.00
	inc	.01*	.03*	.01*	.02*	.00	.00*	.00	.00*
*σ*	dec	-.03*	-.16*	-.02*	-.15*	.01*	-.02*	.01*	-.01*
	inc	.04*	.12*	.05*	.10*	-.01*	.02*	-.01*	.01*
*f*_*κ*_	dec	.03*	-.12*	.05*	-.10*	.01*	-.01*	.01*	-.01*
	inc	.34*	.30*	.24*	.28*	.12*	.15*	.04*	.13*
*f*_*ρ*_	dec	-.04*	-.03*	-.01*	-.01*	.00	-.02*	.00	.00*
	inc	.04*	-.02*	.01	-.01*	.00*	.01*	.00	.00

d/p refer to the respective number of departments and projects, dec/inc show results for decreased/increased values as compared to the baseline scenario.

* denotes statistical significance at the 5% level.

**Table 9:** Changes in PL for changes in individual parameters.

		*p*_*R*, *E*_ = .2				*p*_*R*, *E*_ = .8			
	d/p	2/2	2/8	8/2	8/8	2/2	2/8	8/2	8/8
*T*	dec	-.02*	-.02*	-.02*	-.02*	.00*	.00*	.00	.00
	inc	.01	.02*	.01*	.01*	.00	.01*	.00*	.00*
*σ*	dec	-.15*	-.19*	-.09*	-.16*	-.02*	-.03*	-.01*	-.02*
	inc	.16*	.14*	.08*	.09*	.02*	.03*	.01*	.02*
*f*_*κ*_	dec	-.09*	-.17*	-.08*	-.16*	-.01*	-.02*	-.01*	-.01*
	inc	.16*	.26*	.16*	.28*	.06*	.13*	.06*	.15*
*f*_*ρ*_	dec	-.02*	.01*	-.01	-.01*	-.01*	-.01*	.00*	.00*
	inc	-.02*	-.06*	.01*	.00	.00	.00	.00	.00

d/p refer to the respective number of departments and projects, dec/inc show results for decreased/increased values as compared to the baseline scenario.

* denotes statistical significance at the 5% level.

**Table 10:** Changes in CL for changes in individual parameters.

		*p*_*R*, *E*_ = .2				*p*_*R*, *E*_ = .8			
	d/p	2/2	2/8	8/2	8/8	2/2	2/8	8/2	8/8
*T*	dec	.01	.00*	.00	-.01*	.00	.00	.00	.00
	inc	.00	.01*	.00	.01*	.00	.00	.00	.00
*σ*	dec	.11*	.03*	.08*	.01*	.03*	.01*	.01*	.00*
	inc	-.12*	-.02*	-.03*	.01*	-.03*	-.01*	-.02*	.00
*f*_*κ*_	dec	.11*	.05*	.13*	.05*	.03*	.01*	.02*	.00*
	inc	.18*	.04*	.08*	.00	.07*	.03*	-.02*	-.02*
*f*_*ρ*_	dec	-.03*	-.04*	-.01	-.01*	.01*	.00	.00	.00
	inc	.06*	.04*	.02*	.01*	.00*	.01*	.00	.00

d/p refer to the respective number of departments and projects, dec/inc show results for decreased/increased values as compared to the baseline scenario.

* denotes statistical significance at the 5% level.

The results from the ceteris paribus analysis show that for good general forecasting abilities the absolute value of the loss measures TL, PL and CL mostly remains unchanged. However, for bad general forecasting abilities, individually changing parameters from the set {*T*, *r*_*c*_, *σ*, *μ*, *f*_*κ*_, *f*_*ρ*_} considerably affect results.

## Summary and policy reflections

This paper explores the effect of errors in forecasting measures associated with investment opportunities on the efficient coordination of distributed decision making. In this context the relevant measures are (i) the initial cash outlay necessary to operate an investment opportunity, (ii) the intertemporal distribution of cash flows, and (iii) the departmental efficiency in operating investment projects. We particularly focus on the case of investment opportunities that are jointly operated by at least two organizational departments. For this purpose, we provide a budget allocation mechanism for the efficient coordination of decisions regarding shared investment projects, which is inspired by previous work done by Baldenius, Dutta, and Reichelstein [7] and Leitner and Behrens [10, 21, 23]. We then set up a computational multi-agent model of a stylized firm in order to test the robustness of the proposed mechanism in two regards: (i) the framework conditions in which the mechanism is deployed (in terms of the number of participating departments and the number of investment projects under consideration), and (ii) errors in forecasting the measures associated with investment opportunities.

On the basis of comprehensive simulation experiments, we show that the probability of not to choose the SHV maximizing investment opportunity increases with the number of investment opportunities considered in one round of budget allocation and slightly decreases with the number of departments operating one investment project (if all three investigated measures associated with investment projects are subject to misforecasting). However, the impact of the considered number of investment projects is significantly higher than the impact of the number of departments (cf. Fig. 5). This finding is independent of the departments’ forecasting abilities, although our results indicate that the pattern becomes more pronounced for relatively ‘good forecasters’. Switching the focus from the probability of a suboptimal decision to the monetary effects indicates that the observed pattern is consistent with respect to the total loss in SHV (which includes both, project loss and compensation loss, cf. Fig. 6) and project loss (cf. Fig. 7). For the definition of loss measures cf. Sect. ‘Error measures’. However, the smoothing effect of the number of departments operating one joint project becomes more pronounced in the case of total loss, and is most pronounced if the focus is put on project loss only. For the impact on the departments’ compensation (cf. Fig. 8), we show that the pattern, which is observed for the probability of a suboptimal decision, total loss, and project loss, *substantially* shifts. First of all, we show that *on average* compensation loss is negative irrespective of the departments’ forecasting abilities. We can, thus, refer to it as compensation *gain*, i.e., forecasting errors lead to a decrease in the departments’ compensation and, thus, leads to a gain from the organizations point of view (as compared to error free scenarios). However, the compensation gain reaches it’s highest values for setups with a low number of departments operating one project and a low number of investment projects considered in one round of funding. Recall, in the case of project loss we have observed the smallest loss in SHV in setups with only a few departments operating one joint project and only a few investment opportunities, which are considered in one round of funding. Together with the fact that compensation *gain* is highest in these scenarios, we can observe scenarios, where total loss becomes negative, an thus also can be referred to as *total gain*. This is particularly the case for setups with two projects per round of funding and two departments per project only. This implies important policy advice: given sparse ‘2 projects/2 departments’ setups, a slight extent of misforecasting is warranted, as it positively affects SHV as compared to error free scenarios. For all other scenarios, it appears obvious that the number of investment opportunities considered in one round of budget allocation as well as the number of departments operating one project can be utilized as short-term policy parameters from the firm’s point of view (see also [10]). We refer to the configuration of these two parameters as ‘framework conditions’. From our results, we derive policy advice on how to set framework conditions for the most efficient utilization of the proposed coordination and budget allocation mechanism. In particular, we can primarily advice to aim at decreasing the number of departments considered in one round of funding. Recall, sparse ‘2 projects/2 departments’ setups —on average—lead to a ‘total gain’. Leitner and Behrens [23] suggest to control the number of investment opportunities proposed for funding via a minimum return on investment they have to fulfill. If organizations want to decrement the number of applications for funding in one round of funding in the short run, this appears to be a feasible strategy, as investment opportunities which exhibit a minor extent of profitability remain unconsidered. However, one alternative strategy would be to split the financial resources available for the funding of projects into multiple funds, and ‘play’ some rounds of funding in parallel. One can, for example, imagine that each of these funds has a special dedication. It could, for example, be the case that one fund is dedicated to the funding of (competing) investment opportunities related to research and development, while for another fund only applications for funding related to operating equipment (e.g., machinery) are considered in the allocation of financial resources. As a result, the number of eligible investment opportunities per fund would decrease, as compared to the case where the financial resources are not dedicated investments related to particular aims. Consequently, splitting the available financial resources into funds would also be a feasible strategy to increase the mechanism’s robustness to errors *in the short run*. However, if organizations are unable to decrement the considered number of investment opportunities in one round of funding, we show that the number of departments operating one investment opportunity becomes particularly relevant if the number of considered investment opportunities is high. Thus, we can advice that increasing the number of departments operating one project should be the preferred strategy if decreasing the number of projects is no option. However, recall that this advice leads to a limitation of damage (in terms a loss in SHV) but will never lead to a ‘total gain’. With respect to the cooperation among departments, Song, Montoya Weiss, and Schmidt [22] argue that there is an increased interest of firms to stimulate, facilitate, and maintain cooperation across organizational functions. In this regard, they exemplarily refer to the formation of cross-functional or cross-departmental team building and quality function deployment for the case of product development as methods to enhance cooperation (cf. also [44, 45]). Similarly, Weiss and Hughes [46] provide and discuss strategies for enhancing intra-organizational cooperation. They argue that organizations should rather aim at focusing on the roots of missing collaboration rather than on their consequences. For the long run, [46] provide a financial and a rather non-financial strategy for fostering collaboration: (i) They argue that cooperation among departments can be enhanced by designing an appropriate incentive system. This could, for example, mean that collaborative behavior is rewarded. A similar approach is presented by Baiman and Baldenius [47] who argue that cross-departmental cooperation could be fostered by considering non-financial performance measures in compensation. Such a non-financial performance measure could, for example, be the number of successfully implemented joint projects. They [47] argue that such non-financial performance measures are significantly superior to variable compensation components which are based on firm wide performance (which one could regard as a appropriate performance measure that includes cooperation). Utilizing a firm-wide performance measure would require a lot of communication among departments and a higher compensation risk for department managers [47–49]. From a non-financial perspective, Weiss and Hughes [46] argue that (ii) teaming is an indispensable factor to enhance intra-organizational cooperation (for ‘teaming’ cf. also [50]) and that organizations can be structured for cooperation. In a long-term perspective, one can imagine that both aspects of our preliminary conclusion—the number of project proposals and the number of departments operating one proposal—can be affected via organizational design. With regard to the encouragement of intra-organizational cooperation, Gittel and Weiss [51] provide an overview of approaches such as cross-functional routines or protocols (cf. [52, 53]), information systems (cf. [54]), cross functional boundary spanners (cf. [55]), cross-departmental meetings (cf. [56]), shared incentives, and shared performance measures (cf. [57, 58]). This argumentation is in line with Keidel, Bell, and Lewis [59], who argue that restructuring (manipulating units within organizations), reengineering (revising organizational processes), and rethinking (the way in which issues and decisions within organizations are patterned) are the core of organizational design. They reason that particularly ‘rethinking’ impacts the extent of cross-departmental cooperation and, thus, appears to be of high importance in this context.

When it comes to improving forecasting quality (i.e., decreasing the number of measures that are forecasted with errors), we show that the sequencing of data quality activities is crucial for improving the proposed mechanism’s robustness to errors in the best possible way, and that the framework conditions in which our coordination mechanism is deployed play a crucial role. Recall, by the sequencing of data quality activities we refer to the question of which measures associated with investment opportunities to focus on first. In particular, we provide evidence that for scenarios, in which departments have perfect knowledge on the intertemporal distribution of cash flows solely or in combination with their efficiency in operating investment opportunities or the initial cash outlay necessary to operate the project, respectively (cf. Table 3, scenarios 1, 4, and 5), the extent of total loss decreases irrespective of the framework conditions (cf. Figs. 9 and 10). For the remaining scenarios, we show that whether or not the loss in SHV decreases is strictly dependent on the framework conditions. On the basis of this finding, we can provide comprehensive policy advice. For the case that organizations cannot alter the framework conditions and are willing to invest resources into increasing the forecasting quality of *one* measure associated with investment projects, we can derive the following policy recommendations if forecasting quality is bad (cf. Fig. 9): (i) For scenarios with only a few projects, which are considered in one round of funding and a few departments jointly operating one project, we recommend focussing on the intertemporal distribution of cash flows first, as this leads to the highest relative decrease of lost SHV. If (ii) the number of investment projects considered in one round of funding is high and the number of departments jointly operating one investment project is low, it appears to be a beneficial strategy to focus on the departmental efficiency of operating projects first. Finally, if (iii) both the number of projects considered in one round of funding and the number of departments operating a project are high, organizations are better off if they focus on the initial cash outlay first. It is important to note that deviating from the policy advice in case (i) still leads to an increase in SHV, while deviating from our advice in cases (ii) and (iii) may also lead to an increase in lost SHV. However, if organizations are able to alter their framework conditions (as outlined above), Figs. 9 and 10 provide guidance on how to set framework conditions so that the best possible decrease in lost SHV can be achieved. For situations, in which organizations want to simultaneously increase the forecasting quality of two out of three measures, the highest decrease can be observed when the number of considered projects is high and the number of departments operating one project is low. However, particular attention on the framework conditions must be paid in scenarios, in which the forecasting quality of the intertemporal distribution of cash flows and of the departmental efficiency in operating the project should be increased, because, in this case, wrong framework conditions may lead to an increase in total loss. For good departmental forecasts (cf. Fig. 10), in order to decrease the lost SHV in the best possible way, the number of departments considered in one round of funding should always be high, while—depending on which measure organizations want to focus on first—the number of departments operating one joint project can vary. However, the contour plots in Fig. 10 can be utilized to derive the best possible strategy (as exemplarily outlined on the basis of Fig. 9 for bad forecasters). In Table 6, we list the configurations of framework conditions in which total loss hits its minimum, i.e, the configurations which organizations should aspire

In order to illustrate the dynamics of having perfect knowledge regarding one or two measures associated with investment projects, we provide information regarding the impact of perfect knowledge on one or two out of three measures on project loss in Figs. 11 and 12, and regarding the impact on compensation loss in Figs. 13 and 14. It is important to note that the patterns for project loss basically follow the patterns observed for total loss. However, when focusing on compensation loss, also the departments’ point of view should be considered, as the departments’ utility is contingent on their compensation. By contrast, the maximization of compensation loss is a beneficial strategy for departments because by doing so both the variable compensation and the utility for the departments increase. From the organization’s point of view, decreasing compensation loss appears to be a feasible strategy, as this would inevitably lead to a decrease in lost SHV. Basically, we can observe that compensation loss is maximized if the number of departments considered in one round of funding is high. This is opposing to the policy advice we provided for total loss, where we could always observe the minimum total loss for cases in which the number of projects considered in one round of funding is low. For most cases, the same dichotomy can be observed for the configuration regarding the number of departments operating one joint project. The ‘desired configurations’ from the departments’ point of view are also displayed in Table 6. Thus, beside the configuration of framework conditions to decrease the loss in SHV in the best possible way and the information how to sequence data quality activities so that the loss in SHV decreases most efficiently, we provide evidence for diverging interests between the departments and the organization with respect to the configuration of framework conditions. Above, we described ways to control the framework conditions. To the best of our knowledge, the existing literature does not consider this dichotomy in preferences regarding framework conditions. As a consequence, as soon as departments are aware of these dynamics, this might, for example, have an impact on (i) the departments’ willingness to cooperate and work in teams, (ii) the acceptance of organizational restructuring activities, or (iii) the departments’ willingness to implement organizational structures which foster cooperation. Future research might wishes to consider this.

At the same time, our research is, of course, not without its limitations and, as a consequence, encourages future research. First, we proceed from the assumption that there is no explicit communication between the departments of our simulated firm. Future research may wish to take note of this and build an agent-based model, which includes communication between the departments. Several forms of communication channels are conceivable, which would result in different network structures, e.g., rings, lattices, or fully mashed networks (cf. e.g., [11]). In other contexts, there is evidence that the exchange of local information can increase forecasting quality (cf. e.g., [60]). Testing the impact of communication in such networks on the on the efficiency of our proposed coordination mechanism might be a fruitful option for future research. Moreover, as communication among departments could induce agreements between departments with respect to the project characteristics, which are reported to the coordinating unit, future research could apply a game theoretic approach. Then, utilizing appropriate numerical methods (cf. [61, 62]), the impact of the above mentioned network structures in the context of our proposed coordination mechanism might be investigated by future research. Against the background of communication among departments, future research may wish to design the contracts between the departments and the coordinating unit in a way that makes them collusion proof (cf. [63–65]), so that side-contracting behavior is omitted. Second, we assume all decision making managers are modeled to be risk-neutral. In further developments of the model, different levels of risk-propensity or risk-aversion could be investigated. This would impact the utility function of both the departments and the organization and, thus, have an impact on the modeled agents’ behavior. Given different behavioral patterns, the dynamics of the coordination mechanism might change. Altering the agents’ risk attitude might also be of interest in the context of side agreements due to communication among departments. Third, we assume all departments to make errors at the same level, i.e., we suppose them to have similar forecasting abilities. Future research may wish to take this into account and test if our policy advice holds for situations with even a higher level of heterogeneity among agents. Finally, in the current version of our model, synergies of departmental cooperation in terms of the departments efficiency parameters are not considered explicitly. Since efficiencies directly impact cash flows, further research could model the departments efficiency parameters as a function of the number of departments jointly operating one project.

## Supporting Information

S1 Dataset Dataset part 1. http://repository.aau.at/PONE-D-14-42481/S1_Dataset.mat
(MAT)

S2 Dataset Dataset part 2. http://repository.aau.at/PONE-D-14-42481/S2_Dataset.mat
(MAT)

S3 Dataset Dataset part 3. http://repository.aau.at/PONE-D-14-42481/S3_Dataset.mat
(MAT)

S4 Dataset Description of the the structure of the data provided in S1Dataset–S3 Dataset. http://repository.aau.at/PONE-D-14-42481/S4_Dataset.txt
(TXT)

S1 Sourcecode Code of the simulation model. http://repository.aau.at/PONE-D-14-42481/S1_Sourcecode.m
(M) Click here for additional data file.
